# A proactive genotype-to-patient-phenotype map for cystathionine beta-synthase

**DOI:** 10.1186/s13073-020-0711-1

**Published:** 2020-01-30

**Authors:** Song Sun, Jochen Weile, Marta Verby, Yingzhou Wu, Yang Wang, Atina G. Cote, Iosifina Fotiadou, Julia Kitaygorodsky, Marc Vidal, Jasper Rine, Pavel Ješina, Viktor Kožich, Frederick P. Roth

**Affiliations:** 1 The Donnelly Centre, University of Toronto, Toronto, ON M5S 3E1 Canada; 2 Department of Molecular Genetics, University of Toronto, Toronto, ON M5S 3E1 Canada; 3 Department of Computer Science, University of Toronto, Toronto, ON M5S 3E1 Canada; 4 Lunenfeld-Tanenbaum Research Institute, Mount Sinai Hospital, Toronto, ON M5G 1X5 Canada; 5 Department of Medical Biochemistry and Microbiology, Uppsala University, SE 75123 Uppsala, Sweden; 6 Center for Cancer Systems Biology (CCSB), Dana-Farber Cancer Institute, Boston, MA 02215 USA; 7 Department of Genetics, Blavatnik Institute, Harvard Medical School, Boston, MA 02115 USA; 8 California Institute for Quantitative Biosciences, University of California, Berkeley, CA 94720 USA; 9 Department of Molecular and Cell Biology, University of California, Berkeley, CA 94720 USA; 10 Department of Pediatrics and Adolescent Medicine, Charles University, First Faculty of Medicine and General University Hospital in Prague, 128 08 Praha 2, Czech Republic

## Abstract

**Background:**

For the majority of rare clinical missense variants, pathogenicity status cannot currently be classified. Classical homocystinuria, characterized by elevated homocysteine in plasma and urine, is caused by variants in the cystathionine beta-synthase (*CBS*) gene, most of which are rare. With early detection, existing therapies are highly effective.

**Methods:**

Damaging *CBS* variants can be detected based on their failure to restore growth in yeast cells lacking the yeast ortholog *CYS4*. This assay has only been applied reactively, after first observing a variant in patients. Using saturation codon-mutagenesis, en masse growth selection, and sequencing, we generated a comprehensive, proactive map of CBS missense variant function.

**Results:**

Our CBS variant effect map far exceeds the performance of computational predictors of disease variants. Map scores correlated strongly with both disease severity (Spearman’s *ϱ* = 0.9) and human clinical response to vitamin B_6_ (*ϱ* = 0.93).

**Conclusions:**

We demonstrate that highly multiplexed cell-based assays can yield proactive maps of variant function and patient response to therapy, even for rare variants not previously seen in the clinic.

## Background

Rapid development of high-throughput sequencing technology has made it feasible to sequence the genome of every human. However, for personalized diagnostic surveillance and therapy, timely and accurate methods to interpret the clinical impact of genetic variants are needed. Over 138,000 exomes have been collected in the Genome Aggregation Database (gnomAD) [1, 2] and 4.6 million coding variants have been discovered. Among these discovered coding variants, 99% are rare, having a minor allele frequency (MAF) below 0.5%. Although statistical association methods have identified many common variants that correlate with (and in some cases cause) human disease, correlational methods are typically futile for rare variants. In ClinVar [3], the majority of interpreted missense variants are annotated as “variants of uncertain significance” (VUS) [4, 5].

Diverse computational and experimental methods have been developed to predict the functional impact of rare coding variants. Many computational methods can score all possible missense variants proactively and thus provide supporting evidence for variant interpretation immediately upon variant discovery. However, computational predictions were found to identify fewer than 20% of pathogenic variants when used at stringent thresholds where > 90% of pathogenic variant predictions were correct [6]. At more permissive thresholds that detect 90% of pathogenic variants, fully ~ 30% of pathogenicity predictions were erroneous [6]. More accurate predictions can come from experimentally interrogating the functionality of each variant [6], but this one-at-a-time approach is prohibitively laborious and time consuming. Even where done, these experimental assays have necessarily been reactive, i.e., with results that lag far behind the first clinical presentation.

Variant effect (VE) mapping [7, 8] is a strategy for testing the function of a large number of variants in a single experiment. A VE map provides a look-up table for functionality of coding variants in disease-associated genes, potentially providing strong evidence that can be collected in advance of the first clinical observation of a patient variant, so that it is immediately available to assist clinical variant interpretation [9, 10], meeting a clinical need that is particularly acute for rare and personal variants found via sequencing. Although experimental VE maps generally contain some missing data, a recently published VE mapping framework used machine learning to impute missing data so that, given a critical mass of experimental data, missing values could be filled in with accuracy approaching that of experimental measurements [11].

Human cystathionine β-synthase (CBS) is a cytosolic enzyme that catalyzes the first step in the transsulfuration pathway—condensation of serine and homocysteine to yield cystathionine—thus eliminating the toxic metabolite homocysteine [12]. Through alternative reactions, CBS also produces hydrogen sulfide, a gaseous signaling molecule [13, 14]. CBS forms homotetramers and contains heme as a possible redox sensor and/or folding scaffold, pyridoxal 5′-phosphate (PLP; the active form of vitamin B_6_) as a cofactor necessary for catalytic function, and binds *S*-adenosylmethionine (AdoMet) as an allosteric activator repressing the effect of the C-terminal autoinhibitory domain [15].

Each CBS monomer has a modular structure: An N-terminal heme-binding domain is directly followed by a highly conserved catalytic domain of 311 amino acids (aa) in length, which contains the binding site for PLP, including lysine 119, which forms a covalent bond with the ligand. A short (31 aa) linker connects the catalytic domain to a regulatory domain comprised of two AdoMet-binding motifs [16]. Although the exact conformation in which CBS forms its tetrameric complexes is not yet known, as crystallographic analysis has thus far succeeded only for artificial dimeric structures, a potential model has been described [17]. The majority of the CBS sequence is strongly preserved across a billion years of evolution, with the catalytic domain showing the strongest conservation between human and yeast. The AdoMet-binding regulatory domain is slightly less conserved. While this domain is present in yeast, it is absent in some species such as the worm *Caenorhabditis elegans*. The N-terminal half of the linker that connects the two domains is conserved almost as strongly as the catalytic domain, while the C-terminal half is only conserved across vertebrates. Interestingly, yeast carries an 11 aa insertion in the linker, rendering it approximately one third longer than its human counterpart. Finally, the N-terminal heme-binding motif is only partially conserved in some vertebrates and is not present in yeast.

Classical homocystinuria (MIM #236200) [18] is an autosomal recessive disorder of methionine metabolism manifested by abnormal accumulation of total homocysteine in blood, increased excretion of homocysteine in urine, variably elevated methionine levels in blood, and simultaneous decrease of plasma cystathionine. The disease was discovered in 1962 [19] and soon after was shown to be caused by a deficiency of CBS activity in the liver [20]. Since the identification of the first disease-causing CBS variants [21], several hundred alleles have been identified in homozygous or compound-heterozygous homocystinuria patients [22], many of which have been further genetically and biochemically characterized [23–28], yielding ~ 200 annotated pathogenic variants [3, 29]. About 13% of the variants deposited in the CBS Mutation Database [22] are genomic deletions, frameshift mutations, premature termination codons, or missplicing variants, some of which affect *CBS* mRNA stability via nonsense-mediated decay (NMD) [30], while others affect protein folding or biochemical function. However, the majority of these variants (about 87%) are missense variants. Missense variants may affect catalytic function with only minor conformational changes or, substantially more frequently, lead to misfolding amenable to in vitro correction by chemical chaperones or the presence of cofactors [23–26, 31–34]. Regardless of the underlying molecular mechanism, most pathogenic variants yield substantially decreased or null activity of CBS. By contrast, missense variants in the C-terminal domain are mechanistically intriguing. Characterization of several expressed mutations in crude extracts or after purification revealed normal or supraphysiological activity, abnormal AdoMet regulation, and conformational rigidity [21, 22, 32]. However, the small minority of CBS deficiency patients carrying such mutations exhibit high plasma concentrations of total homocysteine and clinical symptoms indistinguishable from patients with variants in the catalytic domain. Two major forms of this disease have been described. Roughly half of the patients suffer from a severe CBS deficiency which manifests in childhood by lens dislocation (luxation), skeletal abnormalities resembling Marfan syndrome, thromboembolism, and neuropsychiatric problems. This type of disease usually does not respond to vitamin B_6_ treatment; however, early initiation of therapy with low methionine diet and/or betaine in the newborn period prevents most of the clinical complications [35]. The other half of the patients suffer from the milder form of disease, which typically manifests by thromboembolism in adulthood and which responds to vitamin B_6_ treatment [35–37]. Although the clinical efficacy of vitamin B_6_ and its effect on decreasing plasma total homocysteine are well established, the underlying mechanism is still unclear. The possible explanations include increased stability of fully PLP-saturated mutant enzymes and most likely a chaperoning effect of co-translationally present PLP on susceptible mutations [15].

The population frequency of severe early-onset CBS deficiency ranges from 1 in 60000 to 1 in 900000 between countries, and the worldwide birth frequency of clinically ascertained patients was estimated to be 1:122000 [38]. However, homocystinuria may be more frequent in specific populations (1:1800 in Qatar) and molecular epidemiological studies suggest a higher frequency of the adult vitamin B_6_-responsive form [36, 37, 39–42].

Since only early diagnosis and timely therapy can effectively prevent long-term complications in patients with homocystinuria, many newborn screening programs worldwide target CBS deficiency [43]. Screening by determining total homocysteine (tHcy) in dried blood spots is only occasionally performed given the need for a reduction step prior to LC-MS/MS assay and associated costs [42]. Therefore, CBS deficiency is usually sought by screening for elevated methionine concentration and subsequent testing for tHcy [44]. Unfortunately, screening newborns for elevated methionine concentrations misses some vitamin B_6_-non-responsive patients and a large proportion of vitamin B_6_-responsive patients [35, 44]. It has not been shown whether future newborn screening programs based on genome sequencing could improve the early detection of homocystinuria.

Yeast complementation assays can identify pathogenic alleles with high accuracy [6]. The human CBS gene can complement growth defects in *cys4∆* yeast deletion mutants [45, 46], and this assay can also be used to test whether variants are vitamin B_6_-dependent [47–50]. Here we adapt this complementation assay to our recently described VE mapping framework and use it to generate comprehensive functional maps of CBS missense variation with low or high levels of vitamin B_6_. We find that scores from the resulting VE maps can identify functional variation in CBS. Moreover, in an independent patient cohort, patient CBS activity scores derived from the VE map correlate strongly with the age of onset, disease severity, and response of CBS-deficient patients to vitamin B_6_ therapy.

## Methods

### Strains and plasmids

The *Saccharomyces cerevisiae* strain (*MATα cys4Δ::KanMX his3Δ1 leu2Δ0 lys2Δ0 ura3Δ0*), used as a host for the CBS variant library, was derived from the yeast knockout collection [51]. The Gateway destination vector pAG415GAL-ccdB (CEN/ARS-based, *GAL1* promoter, and *LEU2* marker) was purchased from Addgene and served as the yeast expression vector. The *CBS* open reading frame (ORF) clone was obtained from the Human ORFeome v8.1 library [52], corresponding to UniprotKB accession P35520.

### Constructing a codon-randomized CBS variant library

A library of *CBS* variants was constructed using an oligo-directed codon-randomization mutagenesis method (Precision Oligo-Pool based Code Alteration or POPCode) [11]. Details are described below, with some technical advancements that decrease the frameshift mutation rate and thus render the method suitable for mutagenizing larger genes. An oligonucleotide with length between 28 and 38 bases was designed to target each codon in the *CBS* ORF, such that the targeted codon is replaced with an NNK-degenerate codon (a mixture of all four nucleotides in the first and second codon positions, and a mixture of G and T in the third position) using the PopCode oligo suite webtool [11]. The 550 oligos were synthesized then combined into a single equimolar pool. A uracil-doped wildtype template was generated by PCR-amplifying the ORF as follows: A 50 μl PCR reaction was set up containing 25 μl 2X Kapa Uracil+ ReadyMix, 2.5 mM dUTP, 10 μM forward and reverse oligos, and 1 ng template DNA. Thermal cycler conditions are as follows: 98 °C for 5 min, 30 cycles of 98 °C for 15 s, 60 °C for 60 s, and 72 °C for 180 s. A final extension was performed at 72 °C for 5 min. Uracilated amplicon was gel-purified using the 1% agarose gel at 80 V for 90 min, and the bands cut out and purified using a QIAquick Gel Extraction Kit (QIAGEN). The final elution volume was 30 μl TE or ddH_2_O. The mutagenesis oligo pool was phosphorylated as follows: A 50 μl reaction containing 10× PNK buffer (NEB), 300 pmol oligos, 10 mM ATP, and 10 U polynucleotide kinase (NEB) was incubated at 37 °C for 2 h. The reaction was used directly in the subsequent POPCode reaction. The uracil-doped templates were then mixed with the phosphorylated oligonucleotide pool. Oligos were annealed to the template by heating the mixture to 95 °C for 3 min and then cooled to 4 °C. Gaps between annealed oligonucleotides were then filled with KAPA HiFi Uracil+ DNA polymerase followed by nick-sealing with T4 DNA ligase (New England Biolabs; NEB). After degradation of the uracil-doped wildtype strand using uracil-DNA-glycosylase (UDG; NEB), the mutated strand was amplified with attB-site-containing primers and subsequently transferred en masse to a donor vector via the Gateway BP reaction (Thermo-Fisher Scientific) to generate a library of entry clones. To enable yeast expression, the library was further transferred to pAG415-ccdB by en masse Gateway LR reaction and transformed into the *S. cerevisiae cys4Δ* mutant strain. To maintain the complexity of the library, plasmids were purified from > 100,000 clones at each transferring step and ~ 1,000,000 yeast transformants were pooled to form the host library.

### High-throughput yeast-based complementation

The yeast-based functional complementation assay for CBS function has been well established for characterizing individual variants [45, 46, 50]. Details are provided here for high-throughput complementation screening: Plasmids extracted from a pool of > 100,000 *Escherichia coli* clones were transformed into the *S. cerevisiae cys4* mutant strain yielding ~ 1 M total transformants. Plasmids were prepared from two replicates of ~ 1 × 10^8^ cells and used as templates for the downstream tiling PCR (two replicates of non-selective condition). Selective media were made with yeast nitrogen base lacking all vitamins and amino acids (USBiological). All other vitamins except vitamin B_6_ were added at standard concentrations [50] and vitamin B_6_ was supplemented at three different concentrations: 0, 1, and 400 ng/ml. Histidine, uracil, and lysine were added to relieve auxotrophies in the mutant strain, and 2% galactose was used as a carbon source to induce *GAL1*-promoter-driven expression. For each of the three pooled complementation assays (each using a different concentration of vitamin B_6_), ~ 4 × 10^8^ cells were inoculated into a 200-ml selective medium for each of two replicates. In parallel, plasmid expressing the wildtype ORF was similarly transformed to the *S. cerevisiae cys4* mutant strain in selective media. Each culture (with two biological replicate cultures for both the selective and non-selective conditions) was grown to full density (5–6 doublings) while shaking at 30 °C. Plasmids extracted from ~ 1 × 10^8^ of cells of each culture were used as templates for the downstream tiling PCR.

### Detecting variant effects on fitness using TileSeq

For each plasmid library, the tiling PCR was performed in two steps: (i) the targeted region of the ORF was amplified with primers carrying a binding site for Illumina sequencing adaptors and (ii) each first-step amplicon was indexed with an Illumina sequencing adaptor in the second-step PCR. We performed paired-end sequencing on the tiled regions across the ORF in two separate sequencing runs with an average sequencing depth of ~ 2 million reads each. All raw sequencing reads were mapped to CBS using bowtie2 [53] to generate alignment files for both the forward and reverse reads. The tileseq_package software [54] was used to parse the alignment files and count the number of codon changes that had been seen on both strands in the paired-read data. The counts for each mutation in each tiled region were subsequently normalized by the corresponding sequencing depth to obtain an “allele frequency” for that mutation.

### Scoring fitness and vitamin B_6_ remediability

Each sequencing experiment contained not only libraries derived from selective and non-selective pools, but also libraries derived from wildtype amplicons, enabling estimation of the component of each mutation’s observed allele frequency in the pool that was derived from PCR errors during library preparation or sequencing errors. After filtering out variants for which selective or non-selective allele frequencies were lower than a level of three standard deviations above the corresponding (false positive) wildtype allele frequencies, data from equivalent codons for each amino acid change were joined. Then, the allele frequencies observed in the wildtype control libraries were subtracted from the allele frequencies of the non-selective and selective conditions respectively. Then, an enrichment ratio (Φ) was calculated for each mutation based on the adjusted selective- and non-selective-condition allele frequencies.

A maximum a posteriori estimate of the error (*σ*) in each enrichment ratio was derived via a weighted average of the observed variance and the a priori estimate of *σ*, according to the error regularization procedure previously described by Baldi and Long [52]. We used two pseudocounts, so that the observed variance was given weight *n*/(*n* + 2), based on having *n* replicates, and the prior variance was given weight 2/(*n* + 2). The prior estimate of *σ* is based on an overall regression of the coefficient of variation values against sequencing coverage and fitness values.

A fitness score (*s*_MUT_) was calculated for each variant as ln(*Φ*_MUT_/*Φ*_STOP_)/ln(*Φ*_SYN_/*Φ*_STOP_), where *Φ*_MUT_ is the enrichment ratio calculated for each variant, *Φ*_STOP_ is the median enrichment ratio of all nonsense variants, and *Φ*_SYN_ is the median enrichment ratio of all synonymous variants, such that *s*_MUT_ = 0 when *Φ*_MUT_ = *Φ*_STOP_ and *s*_MUT_ = 1 when *Φ*_MUT_ = *Φ*_SYN_. Well-measured variants were selected by applying two filters: The allele frequency in the pre-selection library must be greater than 0.005% (to avoid undersampling) and standard error must be less than 0.2.

A vitamin B_6_ remediability (delta) score was calculated as the difference between fitness scores at high (400 ng/ml) and low (both 0 and 1 ng/ml, with fitness scores averaged due to high agreement between these screens, see the “Results” section for details) vitamin B_6_ concentrations.

To produce a complete variant effect map, missing values were estimated by imputation as previously described [11, 55]. Briefly, the imputation machine learning model was trained on the fitness scores of the experimentally well-covered variants using the gradient-boosted tree (GBT) method [56] as implemented by the XGBoost package [57]. The features used in the model included confidence-weighted averages of other variant scores at the same position, confidence-weighted averages of the scores for the 3 and 4 most similar amino acid changes (according to BLOSUM distance), precomputed PolyPhen-2 [58] and PROVEAN [59] scores, chemical and physical properties of the wildtype and substituted amino acids, and protein structure-related information. Final variant effect maps use scores that were refined using the weighted average of imputed and measured values (weighting by the inverse-square of estimated standard error in each input value).

To estimate agreement with previous individual yeast complementation assay data [50, 60], only well-measured values were used. Of the 40 variants for which our map could be compared against assays made in Mayfield et al., 36, 33, and 35 variants were well-measured for the 0, 1, and 400 ng/ml vitamin B_6_ conditions, respectively. Of 206 variants measured in Wei et al., 179 were well-measured in our study. All other analyses used the final imputed and refined map.

### Classifying vitamin B_6_-remediable and non-remediable variants

Using the fitness score distribution of all synonymous variants as an empirical null distribution, FDR-adjusted *p* values were assigned to all missense variants. The fitness score corresponding to FDR = 5% was determined to be 0.60, so that missense variants for which the upper end of the 95% confidence interval of their fitness scores was less than 0.60 were classified as deleterious variants. Then, for each variant that was deleterious in the low vitamin B_6_ condition, a delta fitness score (high vitamin B_6_ − low vitamin B_6_) was calculated. Using the delta fitness score distribution of all nonsense variants as an empirical null distribution, FDR-adjusted *p* values were assigned to all missense variants and a delta fitness score threshold (0.22, corresponding to FDR = 5%) was used to identify vitamin B_6_-remediable variants. Missense variants for which the lower end of the 95% confidence interval of their delta fitness scores was greater than 0.22 were classified as vitamin B_6_ remediable.

### Relating fitness score and enzyme activity

A previous study [23] measured the enzyme activity of CBS variants expressed in *E. coli*. Of the 27 missense variants with measured activity, the 24 that were well measured in this study were selected to investigate the relationship between our fitness scores and enzyme activity. More specifically, we examined the correlation between our high vitamin B_6_ fitness scores and relative CBS enzyme activity (variant activity divided by wildtype activity) with AdoMet at 37 °C. A Michaelis-Menten curve (of the form *y* = *x*/(*x* + *k*), where *y* is the fitness score, *x* is the relative enzyme activity, and *k* is a constant) was fitted to describe the non-linear relationship between fitness and activity.

### A test set of disease- and non-disease-associated variants

To define a set of disease-associated CBS variants, we considered 86 unique missense variants in the CBS mutation database [22] that were not linked to a second variant in the same allele. We next reviewed the relevant literature, accepting only the 74 disease variants that we considered to be high confidence. Of these, 71 fell into the catalytic domain and 3 fell into the regulatory domain (Additional file 1: Table S1). Obtaining a set of non-disease-associated variants was more problematic, as the ClinVar database contained no missense variants annotated as “benign” and only one annotated as “likely benign.” As a proxy set of non-disease-associated variants, we therefore selected all CBS missense variants deposited in gnomAD [1, 2] which (i) had no annotated disease association or experimental evidence of functional impact and (ii) have been observed in at least two individuals (Additional file 1: Table S1). All CBS variants from gnomAD that met these criteria were rare, with minor allele frequency less than 0.005. The positive and negative reference variants from these sets were then divided into subsets for the catalytic and regulatory domains and analyzed separately.

### Phenotypes from a cohort of homocystinuria patients

All patients have been followed in the Metabolic Center in the Department of Pediatrics and Adolescent Medicine at the General University Hospital in Prague. The clinical, biochemical, and molecular genetic data were obtained during routine care, and patients gave their informed consent for DNA analysis. Plasma CBS activity was measured within a research project after obtaining patient informed consent, which also included consent for publication of clinical, enzymatic, and molecular genetic data (approval of the Ethics Committee 1194/13 S-IV).

To assess the clinical severity and vitamin B_6_ responsiveness of CBS deficiency, we developed a semi-quantitative scoring system based both on tHcy changes after vitamin B_6_ administration and on the need for additional therapy. Non-responsive patients, requiring a low methionine diet and betaine supplementation (regardless of vitamin B_6_ therapy), were assigned a vitamin B_6_ responsiveness score of 1. Partially responsive patients, in need of both large doses of vitamin B_6_ and a low methionine diet, were given the score 2. Fully responsive patients requiring only vitamin B_6_ at a dose above 0.5 mg/kg/day to yield tHcy < 50 μmol/L received a score of 3. Extremely responsive patients, requiring vitamin B_6_ at a dose below 0.5 mg/kg/day to yield tHcy < 50 μmol/L, were given a vitamin B_6_ responsiveness score of 4.

Disease severity was scored according to the presence of typical clinical complications at the time of diagnosis or during follow-up in poorly compliant patients and could not be determined in two patients detected by newborn screening. Patients showing no symptoms at the time of diagnosis (i.e., detected by screening family members of patients with diagnosed CBS deficiency) received a severity score of 5. Patients with mild disease (thrombosis in any vascular bed with no other symptoms) received the score 4. Patients with moderate disease (connective tissue involvement with or without thrombosis) were assigned a score of 3. Those with borderline severity (mild cognitive impairment with good social outcome, regardless of other somatic complications) were given a score of 2. Severe disease patients (having severe neuropsychiatric complications including poor social outcome, regardless of other somatic complications) were defined to have severity score 1.

In this cohort, 8 variants were represented once, 4 variants were represented 2–3 times, 4 variants were represented 6–7 times, and 1 variant (p.Ile278Thr) was represented 20 times. To limit the impact of recurrent variants on our analysis, we iteratively removed the patient with the most common variant X until no variant appeared more than three times in the reduced cohort. To select among multiple patients with variant X, we first preferentially removed patients with variants occurring *in cis* with X (thus favoring retention of patients with one variant per allele) and next preferentially removed patients with a nonsense, frameshift, or unknown variant *in trans* (favoring retention of patients with a non-X missense variant in *trans*, to favor diversity of missense variants in the cohort), and patients carrying nonsense or frameshift variants in both alleles.

### Calculating variant effect scores for patient diploid genotypes

To calculate diploid variant effect scores for each patient, we modeled the fitness scores (which range from 0 for null variants to 1 for wildtype-like fitness) for combinations of *in cis* variants as the product of the individual fitness scores and summed the two allele scores. For example, for a patient with genotype p.[X];[Y;Z], we would assign diploid fitness *ɸ*_diploid_ = *ɸ*_X_ + *ɸ*_Y_*ɸ*_Z_. Where phasing was not confirmed, we assumed variants were in *trans*, as CBS deficiency is a recessive trait and patients clearly exhibited biochemical features and clinical symptoms of the disease.

## Results

**Fig. 1:**
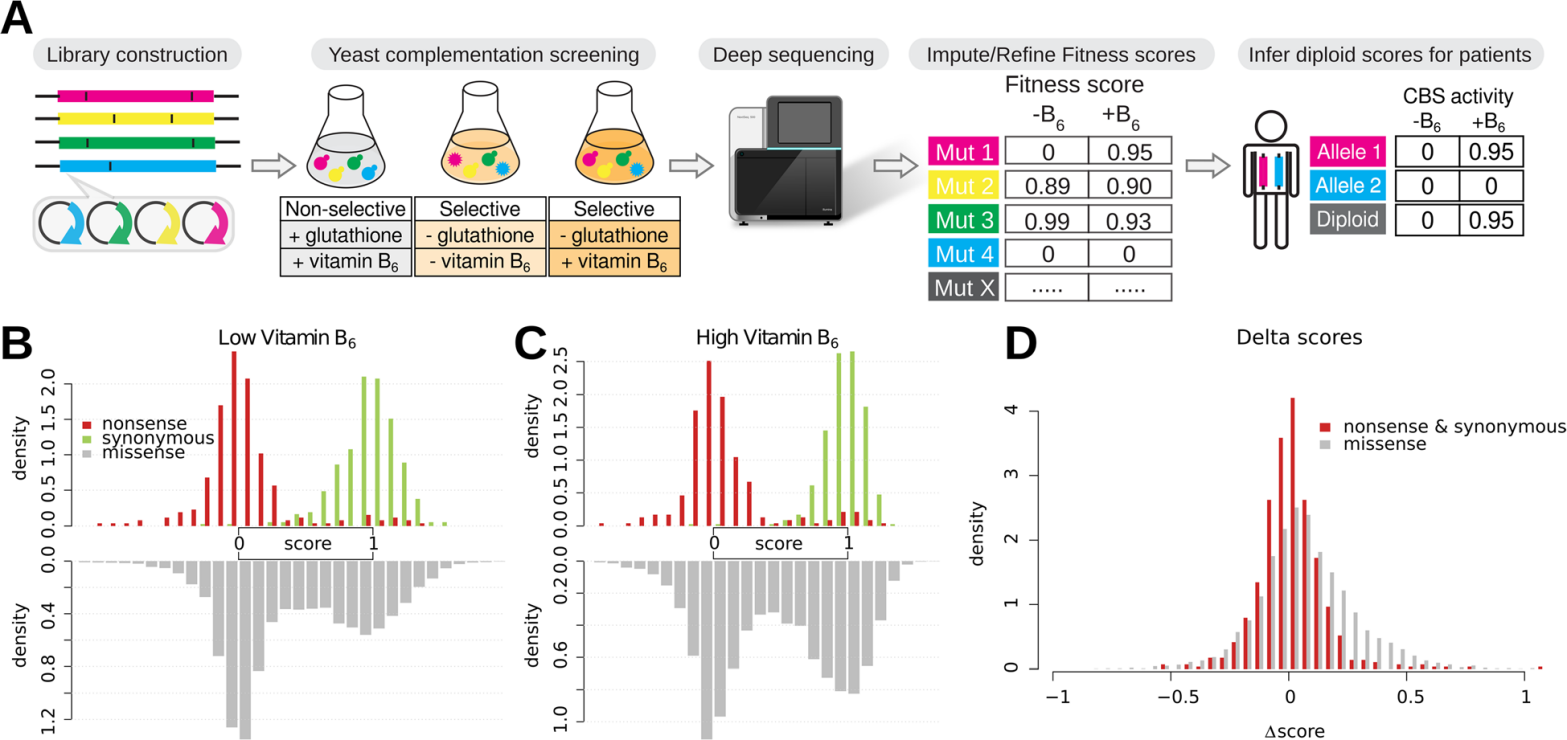
Production of a CBS variant effect map. **a** Workflow for generating the CBS variant effect maps using low or high levels of vitamin B_6_ and inferring total enzyme activities for patients. **b**, **c** Distributions of experimentally determined fitness scores of stop codon, synonymous, and missense variants with low (**b**) or high (**c**) levels of vitamin B_6_. **d** Comparison of the distribution of delta scores for missense variants with the null distribution (delta scores for nonsense and synonymous variants)

To provide a proactive resource to inform the rapid interpretation of genetic variation in CBS, we sought to test all possible missense variants of CBS for functional effects and vitamin B_6_ remediability. We therefore reimplemented a previously validated humanized yeast model [45–48, 50], confirming that expression of human CBS from the hORFeome collection restores the ability of a yeast *cys4∆* strain to grow without supplementation of glutathione (which provides a source for cysteine that circumvents the need for synthesizing cystathionine; see Additional file 2: Figure S1). Coupling this functional complementation with our recently developed framework for exhaustively mapping functional coding variants, we attempted to test the functional impact as well as the vitamin B_6_ remediability of all possible missense CBS variants in parallel (the overall scheme is described in Fig. 1a).

### Proactive maps of missense variant function for CBS

First, we constructed a library of CBS variants using a previously described codon replacement mutagenesis method [11]. The variant library, initially generated as a pool of amplicons, was transferred en masse into the appropriate yeast expression vector via two steps of recombinational subcloning. The resulting library of variant expression clones was then transformed en masse into the yeast *cys4* mutant strain. Sequencing confirmed that mutagenesis resulted in an even distribution of variants across the coding sequence, with the number of amino acid changes per clone following a Poisson distribution with an estimated mean of 2.65 (Additional file 2: Figure S2).

Next, pools of transformed yeast *cys4* mutant strains were grown competitively in selective medium (lacking cysteine and its upstream metabolite glutathione) supplemented with low (0 and 1 ng/ml) or high (400 ng/ml) concentrations of vitamin B_6_. Allele frequencies of CBS variants before and after selection were determined by next-generation sequencing. We used the TileSeq approach [11], sequencing a tiling set of ~ 100 nucleotide segments amplified from the pool. We sought to minimize base-calling errors (which can complicate quantitation of low allele frequency variants within a pool) by sequencing both forward and reverse strands of each template cluster on the flow cell and only accepting variants for which the complementary variant on the opposite strand is also seen. Sequencing was performed such that both forward and reverse strands of each nucleotide position were covered by ~ 2 M reads. In the pre-selection pool, this sequencing detected 83% of all possible missense variants, and 94% of the amino acid substitutions that can be achieved via a single-nucleotide variant (SNV) (Additional file 2: Figure S2C). Fitness scores were calculated for each amino acid substitution based on post-selection changes in allele frequency under both low and high vitamin B_6_ conditions (see the “Methods” section), yielding initial VE maps for CBS. To consider only fitness scores where allele frequencies were high enough to be accurately measured, we kept only the ~ 50% of codon substitutions (corresponding to 75% of amino acid substitutions) with a pre-selection allele frequency above 0.005% (see the “Methods” section, Additional file 2: Figure S2C).

Fitness scores from the resulting VE maps were strongly correlated between replicates (Pearson correlation coefficient (PCC) ranging from 0.86 to 0.94, Additional file 2: Figure S3). Correlation was also strong with the relative growth rates previously determined in single-variant growth assays [50] with PCC values up to 0.8 (Additional file 2: Figure S4A-C). Our results also showed weaker but still-significant correlation with another single-variant analysis [60] (Additional file 2: Figure S4D). Because fitness scores were highly correlated (PCC = 0.97) between the two screens with low levels of vitamin B_6_ (0 and 1 ng/ml), we combined these two datasets to generate a single set of “low vitamin B_6_” fitness scores (Additional file 2: Figure S4E). We also calculated a regularized standard error of each score based on the agreement between replicates as well as a prior informed by sequencing coverage [11, 61]. 97.7% of scores had an estimated regularized standard error of less than 0.2 (Additional file 2: Figure S5). We filtered each map further to consider only scores below this error threshold. After filtering, 59.8% of all possible missense amino acid substitutions and 60% of all SNV-accessible amino acid substitutions were well measured in the low vitamin B_6_ map (Additional file 2: Figure S2C). Similarly, 58.2% of all missense variants and 59.6% of SNV-accessible substitutions were well measured in the high vitamin B_6_ map.

Synonymous variants and nonsense variants each exhibited unimodal fitness score distributions that were well separated from one another (Fig. 1b, c). The separation was slightly more pronounced in the regulatory domain (AUPRC = 0.97) than in the catalytic domain (AUPRC = 0.94) (Additional file 2: Figure S6). Missense variants under both selection conditions showed bimodal distributions (Fig. 1b, c). We also calculated a “delta” fitness score (high vitamin B_6_ − low vitamin B_6_ fitness score) for each variant. The distribution of delta fitness scores for missense variants had a longer positive tail than did nonsense and synonymous variants, indicating that the fitness of some missense variants was substantially increased by elevated levels of vitamin B_6_ (Fig. 1d).

Given a critical mass of experimental variant effect measurements, missing data can be imputed with accuracy approaching that of experimental measurement using a machine learning model [11, 55]. Therefore, we used a gradient-boosted tree regression model [55–57] to impute missing entries and refine variant scores that were measured with lower confidence through weighted averaging (see the “Methods” section). When evaluated using 10× cross-validation, the machine learning prediction achieved a root-mean-squared deviation (RMSD) of 0.28 and a Pearson correlation of 0.64 and 0.63 for the high and low vitamin B_6_ conditions, respectively (Additional file 2: Figure S7A-B). This performance places its quality on par with the experimental dataset itself (given the amount of deviation observed when comparing the high-throughput experimental data against existing low throughput data in Additional file 2: Figure S4A-D). The machine learning method thus allowed for the missing 37% and 39% of VE map scores in the low and high vitamin B_6_ conditions, respectively, to be imputed. Experimentally determined values were refined, using a weighted average between experimental and imputed values, with weighting proportional to measurement confidence. Although this refinement step reduced the number of low-confidence variants (Additional file 2: Figure S7C), the effects of refinement were overall minimal, as 99% of variants had their scores adjusted by less than 0.1 (on the scale from 0 to 1 representing the difference between the average effects of nonsense and synonymous variants) (Additional file 2: Figure S7D). As observed previously [11], the most important features informing the machine learning method were intrinsic to our experimental data: Quality-weighted averages of the 3 and 4 most closely related amino acid changes (according to BLOSUM distance) had the greatest impact, followed by conservation and biochemical information (Additional file 2: Figure S7E). Because imputation is largely derived from averaging experimental measurements for other variants at the same amino acid position, we note that even the imputed scores are, in essence, based on experimental measurements.

**Fig. 2:**
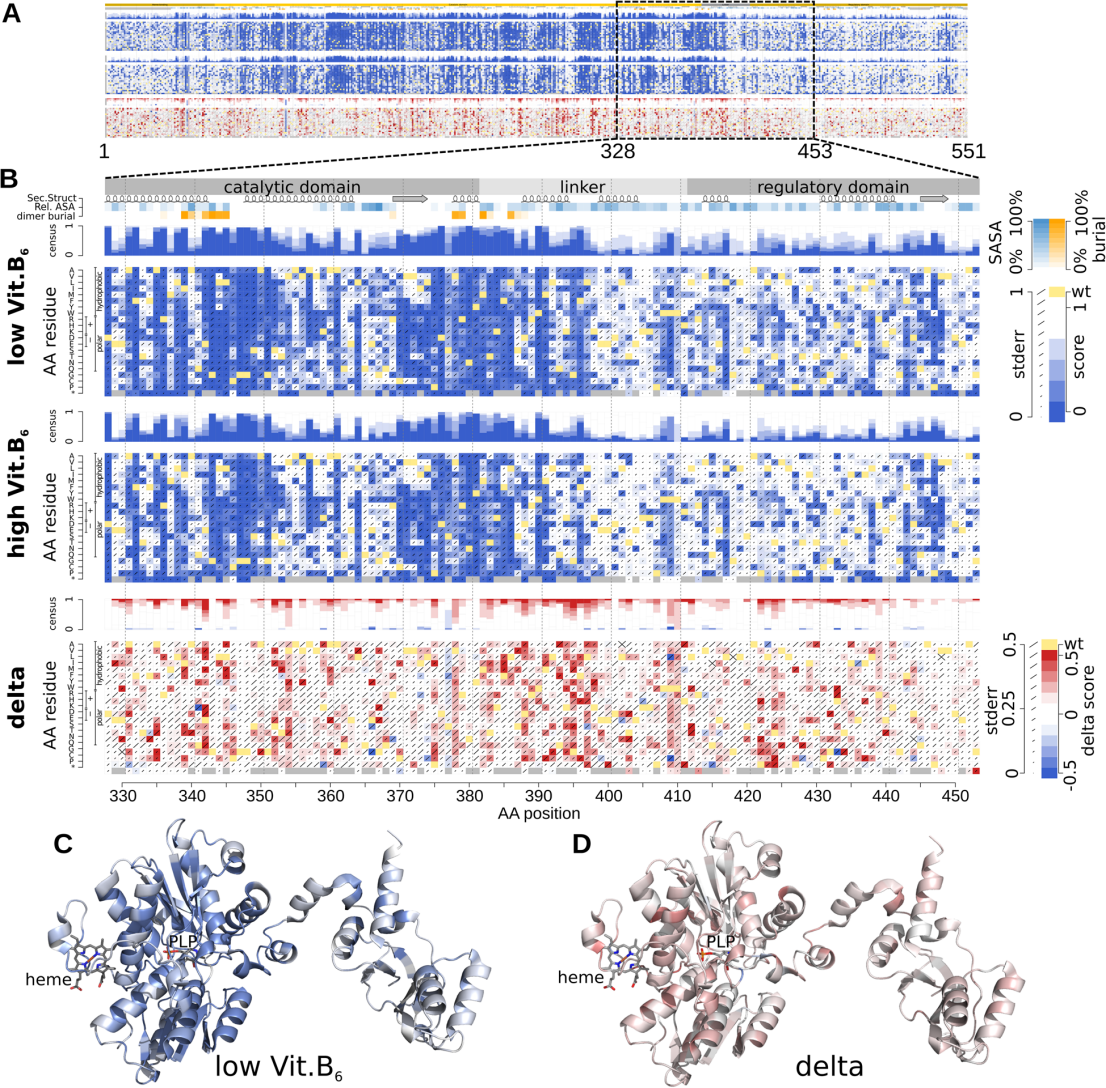
An excerpt of the CBS variant effect (VE) maps and accordingly colorized structures of CBS. **a** A preview of the full map highlighting the region of the cut-out. A poster-sized version of the entire map can be found in Additional file 2: Figure S12. **b** A magnified cut-out of the VE maps for CBS covering amino acid 328–453: fitness landscape with low level (top) and high levels (center) of vitamin B_6_ and the delta fitness (high − low vitamin B_6_) landscape (bottom). In each plot, the first four rows indicate domain annotations, secondary structure, relative solvent accessibility, and burial in quaternary structure, respectively. The next subpanel summarizes the distribution of fitness scores at each amino acid position. The bottom subpanel shows fitness scores for each possible amino acid substitution and nonsense mutation. For high and low vitamin B_6_ VE maps, a functional score of 0 (blue) corresponds to the median fitness of nonsense variants. A score of 1 (white) corresponds to the median fitness of synonymous variants. Yellow fields indicate the wildtype amino acid at each position. Gray fields indicate missing data. Diagonal lines indicate standard error, with crossed out fields marking variants for which standard error exceeded 1. For the delta fitness landscape (high − low vitamin B_6_), substitutions were colored red if delta fitness score is positive and blue if negative. **c**, **d** Crystal structure of a CBS dimer with residues colored according to the median variant fitness with low vitamin B_6_ (**c**) or the median delta fitness score (**d**). The CBS structure shown is based on PDB entry 4L3V [17]

The imputation and refinement procedure yielded complete variant effect maps for CBS under both low and high vitamin B_6_ conditions, which in turn enabled a map of functional remediability of missense variation to different vitamin B_6_ levels (see the “Methods” section; Fig. 2a, b; Additional file 3). For comparison, the pre-imputation version of the maps is shown in Additional file 2: Figure S8.

The “delta” map, measuring high vitamin B_6_ − low vitamin B_6_ fitness, showed that a substantial fraction of missense variants have increased activity at an elevated vitamin B_6_ level. To better understand the mechanisms of vitamin B_6_ remediation, we examined the low vitamin B_6_ map to identify variants with fitness scores that were significantly worse than the fitness distribution of synonymous variants (see the “Methods” section; Additional file 2: Figure S9A). Variants that were deleterious under low vitamin B_6_ conditions were then classified as vitamin B_6_-remediable or non-remediable according to whether their delta fitness score significantly deviated from the distribution of delta scores for nonsense variants (see the “Methods” section; Additional file 2: Figure S9B).

To examine the effects of imputation on delta scores, we separately examined distributions of delta scores for the 64.7% of variants for which neither high nor low vitamin B_6_ score was imputed, the 6.6% where one of the scores was imputed, and the 28.7% where both scores were imputed. The involvement of imputation was associated with an increase in the median delta score, but the size of these effects were minor (effect sizes 0.039 and 0.021 for partially and fully imputed variants, respectively). Moreover, delta scores based on imputed data showed fewer and less extreme outliers (Additional file 2: Figure S10).

Finally, we wondered whether variants that introduce amino acid changes equivalent to the orthologous *S. cerevisiae* sequence showed increased variant fitness due to improved adaptation to the yeast host environment. We therefore compared the set of variants equivalent to *S. cerevisiae*, *C. elegans* (worm) and *Drosophila melanogaster* (fruit fly) residues to a randomly chosen, disjoint set of control variants (Additional file 2: Figure S11). While yeast residues did indeed display a significant increase in median fitness (Mann-Whitney *U* test, *p* = 5.23 × 10^−11^), so did worm and fly residues (Mann-Whitney *U* tests, *p* = 5.14 × 10^−8^ and *p* = 7.21 × 10^−12^, respectively). Moreover, there was no significant difference between the median fitness scores of substitutions to the orthologous yeast, worm, and fly residue. These observations suggest that, while variants seen in the host species are more likely to be tolerated, this effect tends to arise from general functional conservation rather than host adaptation.

### Concordance of maps with biochemical features and enzymatic activity

The set of CBS variant effect maps were largely consistent with known biochemical and structural features of the CBS protein. Early truncating stop codon variants are uniformly deleterious throughout the whole protein except the small linker region between the catalytic domain and the C-terminal regulatory domain. These exceptions are concordant with the previous finding that truncating variants at amino acid positions 409 and 410 increase CBS basal enzyme activity upon expression in yeast by removing the C-terminal autoinhibitory domain [48]; nonsense variants at these positions exhibited slight “hyper-complementation” in the low vitamin B_6_ map (Additional file 2: Figure S8). However, it should be noted that in humans these variants are likely to be subject to nonsense-mediated decay (NMD) and therefore pathogenic [30]. Nonsense variants within the regulatory domain were largely deleterious, consistent with previous observations of such truncations resulting in inactive enzyme [62].

Coloring each residue in the CBS crystal structure with the median variant fitness at that position shows that residues in the central PLP-binding catalytic domain, and especially those nearest to bound PLP, are intolerant to variation (Fig. 2c). Positions in the heme-binding domain are more tolerant to variation compared to the PLP-binding domain (Mann-Whitney *U* test, *f* = 63.68%, *p* = 2 ⨉ 10^−115^, Additional file 2: Figures S12 and S13). However, substitutions of the heme-binding residue His65 are detrimental (Additional file 2: Figures S8 and S12). The C-terminal AdoMet-activated repressive domain is more tolerant to variation (Mann-Whitney *U* test, *f* = 69.30%, *p* < 2.2 ⨉ 10^−16^, Additional file 2: Figures S12 and S13) suggesting that, at least for the yeast strain and growth media conditions we used, the function of this domain does not contribute as much to yeast complementation. Intriguingly, the map also shows a number of variants in the regulatory domain with fitness levels greater than the average synonymous variant. While it is tempting to hypothesize that these variants disrupt the autoinhibitory function of the regulatory domain, there is little biochemical evidence supporting this [24, 25].

**Fig. 3:**
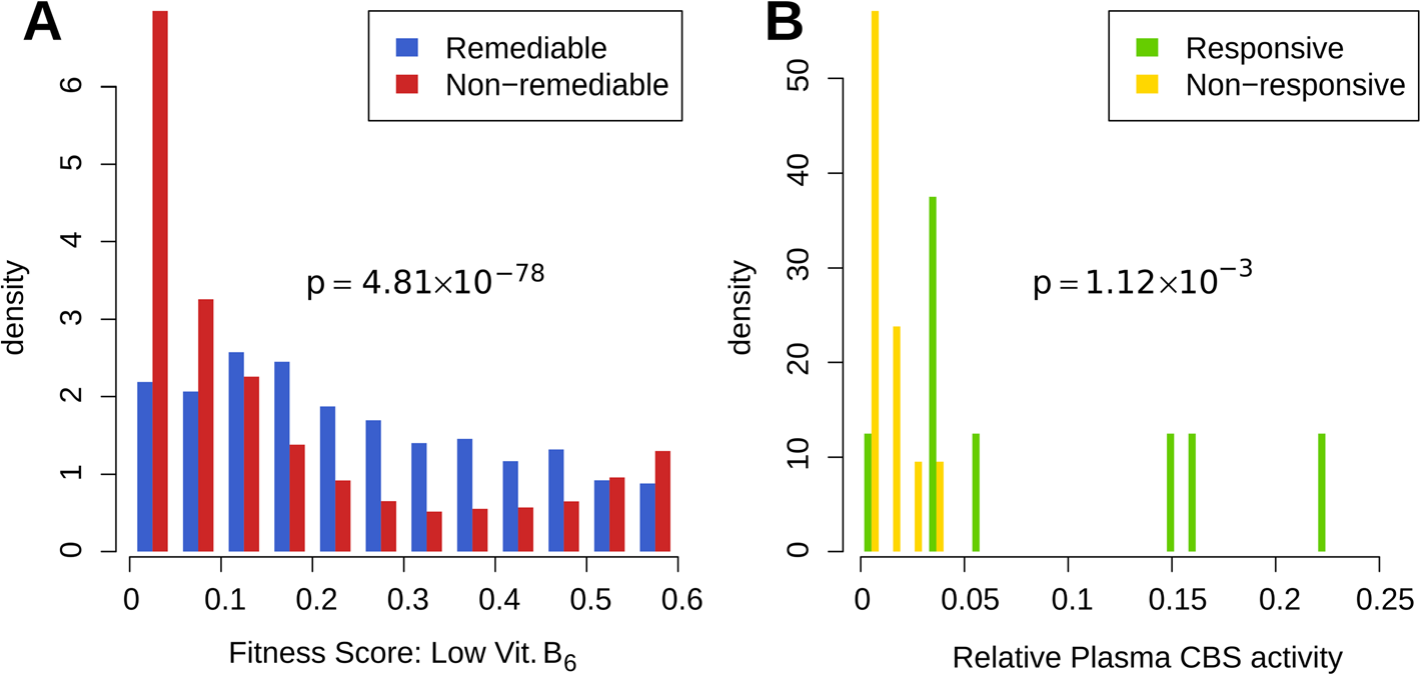
Variant effect maps confirm that vitamin B_6_ is more likely to remediate the weakest effect variants. **a** Distributions of low vitamin B_6_ fitness scores for variants that were deleterious under the low vitamin B_6_ condition, separated according to whether or not they were vitamin B_6_ remediable. **b** Plasma CBS activity distributions of vitamin B_6_-responsive and non-responsive homocystinuria patients (relative to median activity in controls)

The well-documented clinical responsiveness to vitamin B_6_ has not yet been fully elucidated mechanistically, but has been proposed to result from a chemical chaperoning effect [15, 49]. To better understand the mechanistic underpinnings of vitamin B_6_ remediability of human CBS variants in the yeast model, we examined the delta scores resulting from our maps (Fig. 2d) together with multiple features, including: the fitness score itself, computationally-predicted binding energy changes, residue solvent accessibility, and six secondary structure features. Although one might naively think that variants that were the most damaging under the low vitamin B_6_ condition would be easiest to improve, the predicted change in folding energy (∆∆G) tended to be smaller for remediable variants (median ∆∆G was 1.66× higher in non-remediable variants; Wilcoxon test, *p* = 5.61 × 10^−28^; Additional file 2: Figure S14A). Indeed, substitutions with modest fitness scores in the low vitamin B_6_ map were most likely to be vitamin B_6_ remediable: While the median fitness score of non-remediable variants was 0.09, the median score of remediable variants was 0.22 (*p* < 9.63 × 10^−78^), indicating that some residual CBS enzyme activity is required for rescue via elevated vitamin B_6_ (Additional file 2: Figure S14B; Fig. 3a). This result is concordant with clinical observations that 88% of vitamin B_6_-responsive homocystinuric patients have appreciable CBS activity (above 4% that of wildtype; as measured in patient plasma by LC-MS/MS [63]), while only 9.5% of vitamin B_6_-non-responsive patients have appreciable CBS activity (Fig. 3b; Additional file 4: Table S2, see the section “Concordance of CBS maps with pathogenicity and clinical phenotypes” for a discussion of patient phenotype prediction.). We found that positions in a beta-strand secondary structure tended to have lower delta scores, while residues in 3_10_-helices tended to have higher delta scores. Both trends were significant (Wilcoxon test; *p* = 5.45 × 10^−19^ and *p* = 0.02, respectively; Additional file 2: Figure S14C-H) but had small effect size (0.028 and 0.013 difference in median delta scores, respectively). We also found that vitamin B_6_-remediable variants tended to have higher solvent accessibility (median solvent accessibility was 1.6 times higher in remediable variants; Wilcoxon test, *p* = 3.9 × 10^−27^; Additional file 2: Figure S14I). This is consistent with a previous hypothesis by Kopecka and colleagues that solvent-accessible mutations in CBS are more correctable by chemical chaperones including vitamin B_6_ [31]. However, as we previously established [11], solvent accessibility is also strongly correlated with variant fitness scores themselves, so that this correlation may just be the result of common cause.

Based on these results, we examined the known 3D structure of CBS [17] with respect to spatial clustering of amino acid positions for remediable variants. We found that the five amino acids with the highest median delta scores (Ser50, Phe197, Arg266, Ile289, and Pro312) were clustered in a region of approximately 28Å diameter on the joint surface of the catalytic domain, and heme-binding region (Additional file 2: Figure S15). Interestingly, these most-remediable residues are also in proximity to Thr53, which features the lowest median delta score and directly neighbors Cys52, one of the two residues coordinating the heme molecule. The consistently negative delta scores in Thr53 may indicate that the effects of mutations at this residue are exacerbated by higher vitamin B_6_ concentrations. Although we can speculate that the clustering of these residues on a common surface reflects an as-yet-unknown molecular interaction interface, we have no independent evidence for this.

To evaluate the relationship between our fitness scores and the residual CBS enzymatic activity, we examined a previous study reporting in vitro catalytic activities for 26 CBS missense variants expressed in *E. coli* [23], (Additional file 5: Table S3). Our fitness scores exhibited a high rank correlation with measured catalytic activity (Spearman *ϱ* = 0.68), and activity and fitness scores exhibited a non-linear relationship as might be expected from theoretical work by Kacser and Burns on the nature of dominant and recessive alleles [64]. We fit such a curve to relate activity to fitness score (see the “Methods” section; Additional file 2: Figure S16) and it was consistent with the recessive behavior expected for CBS loss-of-function variants. Although this model failed to fit some outliers, the likelihood of the data under this fitted model was 2.7 ⨉ 10^11^ times greater than the best possible linear fit.

### Concordance of CBS maps with pathogenicity and clinical phenotypes

**Fig. 4:**
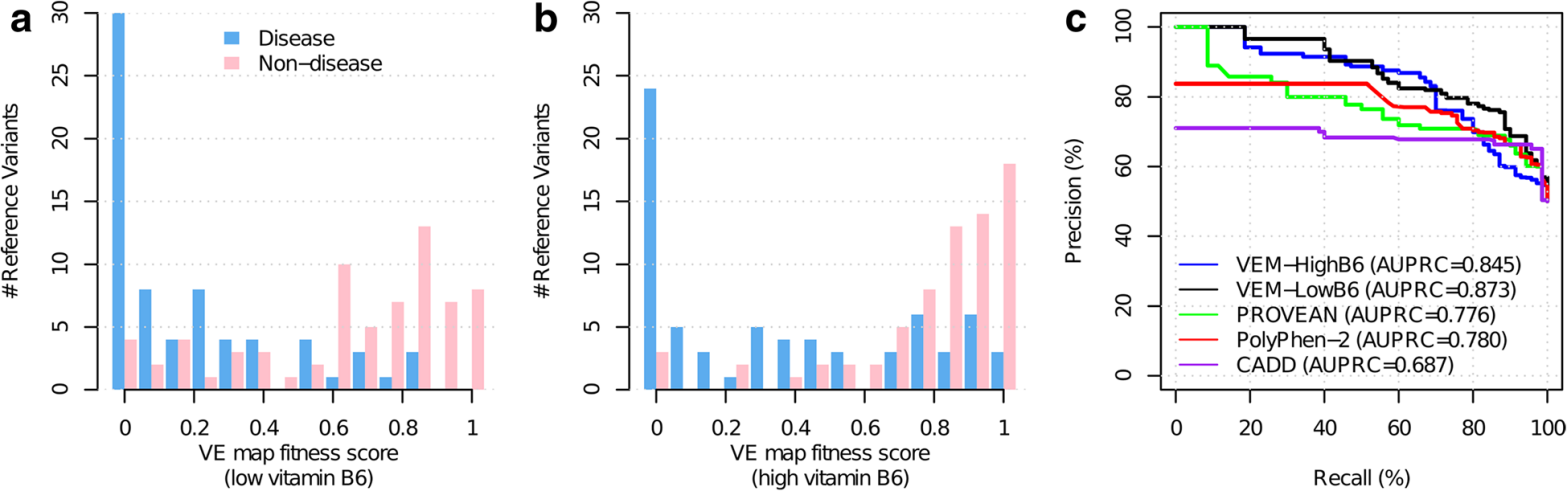
CBS variant effect maps (especially the low vitamin B_6_ map) can successfully distinguish annotated disease-causing variants from other random “non-disease” variants. **a**, **b** Fitness score distributions of disease and non-disease variants with low (**a**) or high (**b**) levels of vitamin B_6_. **c** Precision-recall curves for VE map fitness scores and the computational predictors PROVEAN, PolyPhen-2, and CADD capturing ability of each to discriminate disease from non-disease alleles. VE maps detect many more disease-causing variants at high precision stringency than do any of the computational methods

We next assessed the potential value of our variant effect maps in identifying pathogenic CBS alleles, in terms of the trade-off between precision (fraction of predicted pathogenic variants that are annotated pathogenic) and recall (fraction of all annotated pathogenic variants that were correctly predicted). Because of the generally modest fitness scores in the C-terminal regulatory domain, we examined CBS alleles in the catalytic and regulatory domain separately. A set of 74 high-confidence disease-associated missense variants from the CBS mutation database [22] and 99 rare variants from gnomAD [1, 2] were collected to evaluate prediction performance (see the “Methods” section; Additional file 1: Table S1). However, only 3 of these 74 disease variants were located in the regulatory domain, making its evaluation more difficult. In the catalytic domain, distributions of fitness scores, plotted separately for disease and non-disease alleles, clearly show that fitness scores from both low and high vitamin B_6_ maps can distinguish pathogenic variants (Fig. 4a, b). We then compared the performance in terms of area under the precision vs recall curve (AUPRC) for our two maps with each of three computational methods (PolyPhen-2, PROVEAN, and CADD) [58, 59, 65]. Both of the variant effect maps (AUPRC = 0.84 for high vitamin B_6_; AUPRC = 0.87 for low vitamin B_6_) outperformed all three computational methods (AUPRC = 0.78 for PolyPhen-2; AUPRC = 0.78 for PROVEAN; AUPRC = 0.69 for CADD) (Fig. 4c). At 90% precision, the low vitamin B_6_ variant effect map captured 41% of pathogenic variants, while the best-performing computational method, PROVEAN, captured only 11% of pathogenic variants. These results essentially agreed with our previous study of variants in a panel of 21 human disease genes, which found that yeast complementation assays tended to detect pathogenic variation with triple the sensitivity of the best computational methods [6]. We also evaluated our maps’ performances in the regulatory domain, finding performance that was lower than that of our maps in the catalytic domain, but higher than computational methods in the regulatory domain (AUPRC = 0.40 for the low vitamin B_6_ map; AUPRC = 0.54 for the high vitamin B_6_ map; AUPRC = 0.32 for PolyPhen-2; and AUPRC = 0.31 for PROVEAN; Additional file 2: Figure S17). However, no strong conclusions should be drawn from this analysis of the regulatory domain, given that only three disease variants in this region are known.

We next wished to test whether performance differed between purely experimentally determined variant effect scores and those that were imputed or refined using our machine learning method. We re-calculated performance separately for experimental scores (finding AUPRC = 0.836), for imputed values (AUPRC = 0.856), and for refined values (AUPRC = 0.842; Additional file 2: Figure S18). Imputed scores slightly exceeded the performance of experimental scores, which can perhaps be understood by the fact that these scores are largely driven by averages of other experimental measurements at the same amino acid position. However, the performance of experimental, imputed, and refined scores was numerically quite similar.

Evaluation against positive and negative reference variants allowed us to re-state each variant’s fitness score in terms of a likelihood ratio of pathogenicity. That is, by examining the distribution of fitness scores in the two reference sets, we could determine, for each possible variant, the likelihood of observing a score at least as low in the positive reference set, as well as the likelihood of observing a fitness score at least as high in the negative reference set (Additional file 2: Figure S19A). The ratio of these two likelihoods (also known as a Bayes Factor) expresses how much more (or less) likely the variant is to belong to the positive (presumed disease causing) set rather than the negative (presumed benign) set. Using this strategy, we calculated log likelihood ratios (LLRs) for each variant (Additional file 6: Table S4). The overall distribution of LLRs across all possible amino acid changes in CBS (Additional file 2: Figure S19B) shows that 39% of variants are at least 10× more likely to be pathogenic than benign, while 33% of variants are at least 10× more likely to be benign than disease causing. CBS variant LLRs also range further into the negative values than into positive values: While we found 15% of variants to be >100× more likely to be benign than disease causing given the map evidence, no variants were found to be >100× more likely to be pathogenic than benign.

Finally, we wished to examine the ability of our maps, based on complementation phenotypes in yeast, to predict quantitative human phenotypes. For this purpose, we examined an evaluation cohort of 29 well-phenotyped homocystinuria patients (for genotypes and phenotypic characteristics see Additional file 2: Table S6). Among these patients, 12 were vitamin B_6_-non-responsive, 12 fully or extremely responsive, and 5 partially responsive. Consistent with the established inheritance pattern, all patients were either homozygous or compound heterozygous for CBS mutations. Two additional patients, each carrying an allele in the regulatory domain (p.Trp409*; p.Asp444Asn), were not evaluated because the yeast complementation assay did not appear sufficiently sensitive to perturbation in the regulatory domain (see details in the “Discussion” section). Of the 29 remaining patients, 20 had a genotype involving the allele p.Ile278Thr, thus introducing a potential bias from a single recurrent variant. Therefore, we used an objective protocol to iteratively eliminate patients from the analysis to limit the recurrence of individual variants (see the “Methods” section and Additional file 7: Table S5).

**Fig. 5:**
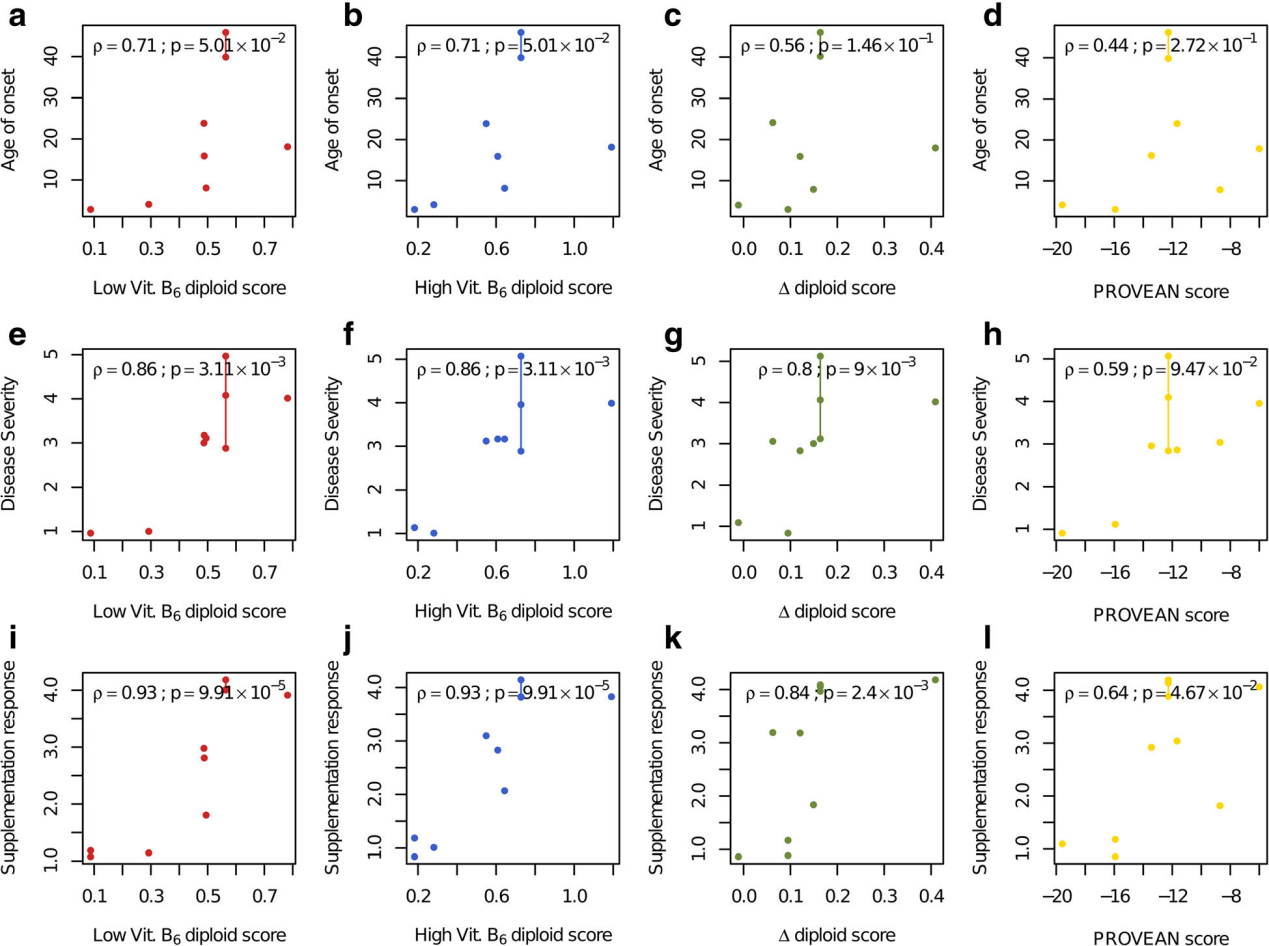
CBS VE maps, which have not been trained on patient data, successfully predict patient phenotype and response to vitamin B_6_ therapy. **a**–**d** Correlation between diploid VE map or PROVEAN scores and age of onset. **e**–**h** Correlation between diploid VE map or PROVEAN scores and disease severity scores. **i**–**l** Correlation between diploid VE map or PROVEAN scores and clinical vitamin B_6_ response. Degree of disease severity: 5 = no symptoms at the time of diagnosis, 4 = mild disease, 3 = moderate disease, 2 = borderline severity, 1 = severe disease. Degree of vitamin B_6_ responsiveness: 1 = non-responsive, 2 = partial responsive, 3 = fully responsive, 4 = extremely pyridoxine responsive. A small amount of random noise (jitter) was added to the categorical values of disease severity and vitamin B_6_ responsiveness to visually separate coincident data points. The amount of random noise is uniformly distributed in the interval [0;0.2]. Vertical lines connect data points with identical genotypes

Based only on a list of the remaining alleles (blinded to phenotypes), we first retrieved each allele’s imputed low and high vitamin B_6_ variant effect map score and calculated diploid scores for each patient by treating variant fitness scores as additive in *trans* and multiplicative *in cis* (see the “Methods” section for more details). Three patient CBS activity scores were calculated, corresponding to the low vitamin B_6_, high vitamin B_6_, and differential (high − low vitamin B_6_) maps. Correlation was examined for each type of patient activity score between each of three clinical phenotypes: age of onset, disease severity, and clinical response to vitamin B_6_ (Fig. 5).

Patient scores derived from the low vitamin B_6_ map yielded strong correlations with patient phenotypes that were highly significant: Correlations in terms of Spearman’s *ϱ* were 0.71 (*p* = 0.05), 0.86 (*p* = 0.003), and 0.93 (*p* = 9.91 × 10^−5^) for age of onset, disease severity, and clinical vitamin B_6_ response, respectively. The high vitamin B_6_ CBS scores correlated just as strongly, with Spearman’s *ϱ* = 0.71 (*p* = 0.05), 0.86 (*p* = 0.003), and 0.93 (*p* = 9.91 × 10^−5^) for age of onset, disease severity, and vitamin B_6_ responsiveness, respectively. The differential (high − low vitamin B_6_) diploid scores showed a weaker correlation at *ϱ* = 0.56 (*p* = 0.14), 0.8 (*p* = 0.009), and 0.84 (*p* = 0.002) for age of onset, disease severity, and vitamin B_6_ responsiveness, respectively. All map-based scores performed better than the computational method PROVEAN [59], which in the previous analysis was the best-performing computational method for pathogenicity classification (see above). PROVEAN yielded very poor correlations of *ϱ* = 0.44 (*p* = 0.27), 0.59 (*p* = 0.09), and 0.64 (*p* = 0.05) for age of onset, disease severity, and clinical vitamin B_6_ response, respectively. In the interest of completeness, an unfiltered version of this analysis with all 29 patients (which may heavily be skewed by our performance for recurrent variants) is also shown in Additional file 2: Figure S20.

In summary, variant effect maps based on experimental measurements of the growth of yeast cells expressing human CBS gene variants, without any further computational fitting or calibration based on human traits, yielded diploid scores that strongly correlated with clinical phenotypes in patients with classical homocystinuria.

## Discussion

Here we generated proactive maps of the effects of missense variation in the human *CBS* gene. Using codon-randomizing mutagenesis to generate a clone library bearing nearly 80% of all possible amino acid changes, we measured the functional consequences of CBS variation by measuring the effects of selection on allele frequencies during a competitive yeast complementation assay using next-generation sequencing. The resulting proactive variant effect maps agreed closely with the results of single-variant assays, and the map for low vitamin B_6_ levels showed especially high performance in identifying pathogenic variants.

A machine learning model was used to impute missing data and refine the maps, with performance in identifying disease variants that was on par with (even slightly exceeding) direct experimental measurement. Although this was initially surprising, it is perhaps more intuitive when one considers that imputation was largely driven by averages of experimental measurements of other substitutions at the same amino acid position.

Overall, we found that our CBS variant effect map could accurately distinguish annotated pathogenic variants from unannotated variants. At a stringent threshold achieving 90% precision in our test set, the variant effect map captured more than twice the number of pathogenic variants than did the best-performing computational prediction method at the same 90% precision stringency.

An important caveat to our maps is that, because the underlying complementation assay is based on expression of mature cDNA, they cannot detect the impact of variants on splicing. Also, some pathogenic variants such as p.Trp409Ter, which in humans would be subject to NMD, were not detected as damaging in our assay. Furthermore, our assay measures protein function in the context of the cellular machinery in the yeast host. Therefore, the assay can miss the functional impact of variants that perturb molecular functions that are important in human cells but not relevant in yeast. In the case of CBS, the function of the AdoMet-binding regulatory domain appeared less important than the catalytic domain to functional complementation, as variants in this domain were generally more likely to be tolerated in our assay. Therefore, our assay may be unsuitable for detecting some pathogenic variants in this regulatory domain (e.g., p.Asp444Asn). Further complicating variant interpretation, a number of variants in the regulatory domain have previously been observed to render CBS biochemically hyperactive and yet paradoxically cause symptoms typical for CBS deficiency [15, 24–26, 63]. Nevertheless, most truncating variants falling within the regulatory domain did behave like null variants suggesting that our assay can still capture some large-effect variants in this domain. Given the uncertainty, however, we excluded CBS alleles in the C-terminal regulatory domain when evaluating the ability of our maps to infer patient phenotypes.

In addition to systematic error, our measurements are also subject to random error. We previously evaluated this aspect of the methodology [11]. Briefly, a relationship exists between the frequency of the variant in the library (as measured by read count) and the magnitude of noise potentially affecting the measurement. An analysis of this relationship was incorporated into a Bayesian error regularization procedure (described in the “Methods” section) to improve our error estimates. Another source of random noise was introduced by the different *in cis* genotypic backgrounds in which each variant can appear. TileSeq measures the log of the ratio of each variant’s marginal frequency (i.e., neglecting *in cis* variant context) in the selective condition relative to its marginal frequency in the non-selective condition. These log-ratio scores are subsequently calibrated using the distribution of log-ratio scores of synonymous variants, so that accurate scoring requires that *in cis* variants will, on average, have the same effect on log-ratio scores of the variant of interest as they will on synonymous variants. Therefore, noise will rise as the fraction of clones with *in cis* variants rises, as the fraction of *in cis* variants with functional effects rises, and fall as the diversity of *in cis* variants rises. To efficiently convey these caveats to the user, we provided estimates of uncertainty for experimental, imputed, and refined map scores and for the Bayes’ factors (LLRs) that we provided to incorporate our results into a Bayesian framework for variant interpretation.

There are also important caveats in the variant sets we used for evaluations. Given the lack of rare missense variants that have been annotated as “likely benign” or “benign” in ClinVar, we instead used gnomAD variants (after excluding known or suspected pathogenic variants) as a negative reference set. Although it cannot be guaranteed that all underlying individuals are indeed symptom-free, variants in this set can at least be expected to be strongly enriched for benign cases, rendering it an acceptable choice in the absence of alternatives.

Another caveat for our predictions of pathogenicity, which applies more broadly to all clinical annotations of variant pathogenicity, is that variants established as pathogenic in one context may not be pathogenic in every patient. This could stem from the recessive nature of a trait or from sources of incomplete penetrance or variable expressivity such as environmental effects, stochastic developmental effects, or modifier alleles. To partially address only the issue of recessiveness, we used our maps to score diploid genotypes of patients with homocystinuria (Fig. 5).

Despite the inherent challenges of predicting clinical phenotypes in diploid humans, our patient CBS activity scores, derived from variants that had been individually assessed in a haploid yeast model, correlated significantly with age of disease onset and with disease severity. Although these correlations were not perfect, they should be considered in the context that different patients who are homozygous for the same variant exhibit wide phenotypic expression [66]. Moreover, the need to reduce bias from recurrent variants meant that our correlations were based on observations from only 7 unique diploid genotypes (involving 11 unique haploid genotypes) from an original set of 29 patients. Given these challenges, we consider it to be surprising that we achieved significant correlation with patient phenotypes for all three phenotypes from measurements in yeast, and especially noteworthy that map-derived activity scores could strongly predict patient responsiveness to vitamin B_6_ supplementation. Performance might be improved further by testing allele combinations in a compound-heterozygous diploid model system.

We made many observations that could be explored further in the future. For example, the variants in the regulatory domain which appeared to grow faster than the wildtype control in the complementation assay. It is conceivable that some of these variants interfere with the autoinhibitory function of the domain and thus increase the biochemical activity of CBS. However, as mentioned above, biochemical hyperactivity due to loss of autoinhibition may have unexpected physiological consequences.

Despite the ability of cell-based complementation assays to detect deleterious variants with high accuracy, additional context will be required to explain the mechanism of defects. For example, it is unclear whether protein function has been reduced due to a direct reduction in enzymatic activity, disruption of the ability to receive an activating modification, or due to misfolding that reduces stability and leads to a lower steady-state protein expression level. There is now ample precedent for VE maps that measure the effect of variation on “sub-functions” such as protein-protein interaction (which might include tetramerization for CBS), protein abundance, or post-translational modification [5, 9, 67].

The clinical complications of CBS deficiency can be reduced dramatically if the diagnosis is made shortly after birth and if treatment is started in early infancy [35]. Many cases of CBS deficiency can be identified through population-level screening in newborns based on methionine levels and/or methionine/phenylalanine ratios which are usually elevated in severe vitamin B_6_-non-responsive forms of homocystinuria [43, 44]. Unfortunately, the majority of vitamin B_6_-responsive patients assessed in newborn screening programs for elevated methionine are missed due to the absence of this biochemical trait [43, 44]. Although CBS deficiency can be diagnosed later in childhood upon presentation with classical (and irreversible) symptoms of lens dislocation, skeletal abnormalities, thromboembolism, and cognitive impairment, many vitamin B_6_-responsive patients do not present until adulthood [35].

The CBS VE maps we provide could have future value in at least three clinical scenarios. First, the ever-growing use of sequencing in routine genetic care may identify variants of uncertain significance (VUS) in symptomatic patients for whom homocystinuria is not strongly suspected (e.g., in patients with cognitive defects or connective tissue disorders, for which many causes are possible a priori). In this scenario, genomic sequencing coupled with the VE map could sensitively detect deleterious *CBS* variants and thus trigger tHcy measurement and further confirmatory testing, while reducing false positives.

The second scenario for potential clinical use involves patients with suspected CBS deficiency and grossly elevated homocysteine levels. In these patients, *CBS* gene sequencing may identify variants that are unclassified or currently classified as VUS. Because elevated homocysteine can be caused by a number of factors [68] and enzymatic confirmation of CBS deficiency is available in only a few laboratories worldwide, functional information from the VE map could enable clinical interpretation of pathogenicity and thus aid in clinical diagnosis.

Third, should population-level newborn genome sequences become available in the future, genome interpretation using the CBS variant effect map has potential value, even in the absence of elevated methionine or early childhood symptoms. Interpretation of detected *CBS* variants, informed by the VE map, could potentially trigger tHcy measurement. Subsequent detection of elevated tHcy could, with further confirmatory testing, identify additional cases of CBS deficiency, particularly those that would be most responsive to vitamin B_6_ therapy (Additional file 2: Figure S21).

There are 497 human genes that encode a cofactor-dependent enzyme, of which at least 193 (39%) reportedly harbor disease-causing variants [69, 70] (Additional file 8 : Table S6). Based on overall rates of missense variation [71, 72], we might expect every individual to carry roughly 5–10 missense alleles in these enzymes on average. We recently performed a survey of assayable genes [11], finding that 53% of genes have assays tractable for VE mapping and ~ 10% have a yeast complementation assay.

## Conclusions

Our study provides a blueprint for systematic proactive experimental evaluation of missense variant effects on human enzymes using cell-based models, including experimental modeling of how the impact of variants depends on therapeutic context. Our study also offers an example of how variant effect maps derived from cell-based assays can be exploited to estimate quantitative human phenotypes and therapeutic outcomes from personal genome information.

## Supplementary information

**Additional file 1: Table S1.** Selected CBS variants and prediction scores for evaluation of prediction performance.

**Additional file 2: **Contains Supplementary **Figures S1-S21**.

**Additional file 3.** DMS data. Contains all raw read counts, experimental scores, and the final imputed and refined data for each variant in each condition.

**Additional file 4: Table S2.** Plasma CBS activity in vitamin B6 responder and non-responder.

**Additional file 5: Table S3.** Relative in vitro catalytic activity for 24 CBS missense variants expressed in *E. coli* and their corresponding high vitamin B6 VE map fitness scores.

**Additional file 6: Table S4.** Log likelihood ratios of pathogenicity and delta scores.

**Additional file 7: Table S5.** Patient clinical phenotypes and vitamin B6 responsiveness, and inferred activity from VE maps.

**Additional file 8: Table S6.** List of enzymes containing cofactors.

## Data Availability

The full data including raw counts, experimental scores, and imputed and refined scores is available on MaveDB, under accession urn:mavedb:00000005-a. A spreadsheet representation can also be found in Additional file 3. A spreadsheet with pathogenicity log likelihood ratios and delta (“remediability”) scores can be found in Additional file 6: Table S4. The TileSeq sequence analysis package (used to calculate relative read frequencies in the pre-and post-selection libraries) can be found on github at https://github.com/rothlab/tileseq_package. The MAVE scoring function can be found on github at https://github.com/jweile/tileseqMave.

## Abbreviations

aa: Amino acids
AdoMet: *S*-Adenosylmethionine, substrate for many cellular methylation reactions and binding partner for the CBS regulatory domain, also abbreviated as SAM
AUROC: Area under the precision-recall curve
CBS: Cystathionine β-synthase
C-terminus: Carboxyl-group terminus of a protein sequence
*CYS4*: Cysteine auxotrophy gene 4, the yeast orthologue of CBS
dNTP: Deoxy-ribonucleoside 5′-triphosphate
dUTP: Deoxy-uridine 5′-triphosphate
*E. coli*: *Escherichia coli*
FDR: False discovery rate, the fraction of positive reports that are incorrect
GAL1 promoter: Galactose-inducible promoter sequence (originating from the *GAL1* gene)
GBT: Gradient-boosted trees, a machine learning algorithm
gnomAD: Genome Aggregation Database
LC-MS/MS: Liquid chromatography followed by tandem mass spectrometry
MAF: Minor allele frequency, the frequency of a given minor allele
*MATα cys4Δ::KanMX**his3Δ1 leu2Δ0 lys2Δ0**ura3Δ0*: Yeast strain of mating type alpha, carrying a kanamycin resistance cassette that replaces the CYS4 locus, as well as carrying null alleles for *LEU2*, *LYS2* and *URA3*, which convey auxotrophies to leucine, lysine, and uracil
mRNA: Messenger ribonucleic acid
NGS: Next-generation sequencing technology
NMD: Nonsense-mediated decay
NNK: Degenerate codon consisting of two random nucleotides (A/C/G/T) followed by a single keto-nucleotide (G/T)
N-terminus: Amino-group terminus of a protein sequence
OMIM: Online Mendelian Inheritance in Man, a database of Mendelian disorders
ORF: Open reading frame, a nucleotide sequence that stretches form a start to a stop codon.
PCC: Pearson’s correlation coefficient
PCR: Polymerase chain reaction
PLP: Pyridoxal 5′-phosphate, the active form of vitamin B_6_
POPCode: Precision Oligo-Pool based Code Alteration, a mutagenesis protocol
RMSD: Root-mean-squared deviation, a metric of prediction error
*S. cerevisiae*: *Saccharomyces cerevisiae*
SNV: Single-nucleotide variant
tHcy: Total homocysteine
UDG: Uracil-DNA-glycosylase
VE map: Variant effect map
VUS: Variant of uncertain significance
WT: Wildtype
