# Supplementary material for: A proactive genotype-to-patient-phenotype map for cystathionine beta-synthase
Source: Genome Med. 2020 Jan 30;12:13. doi: 10.1186/s13073-020-0711-1 (PMC6993387; doi:10.1186/s13073-020-0711-1)
Supplement: Supplementary file 2 — Additional file 2: Contains Supplementary Figures S1-S21. [file 13073_2020_711_MOESM2_ESM.pdf]

Supplementary Figures

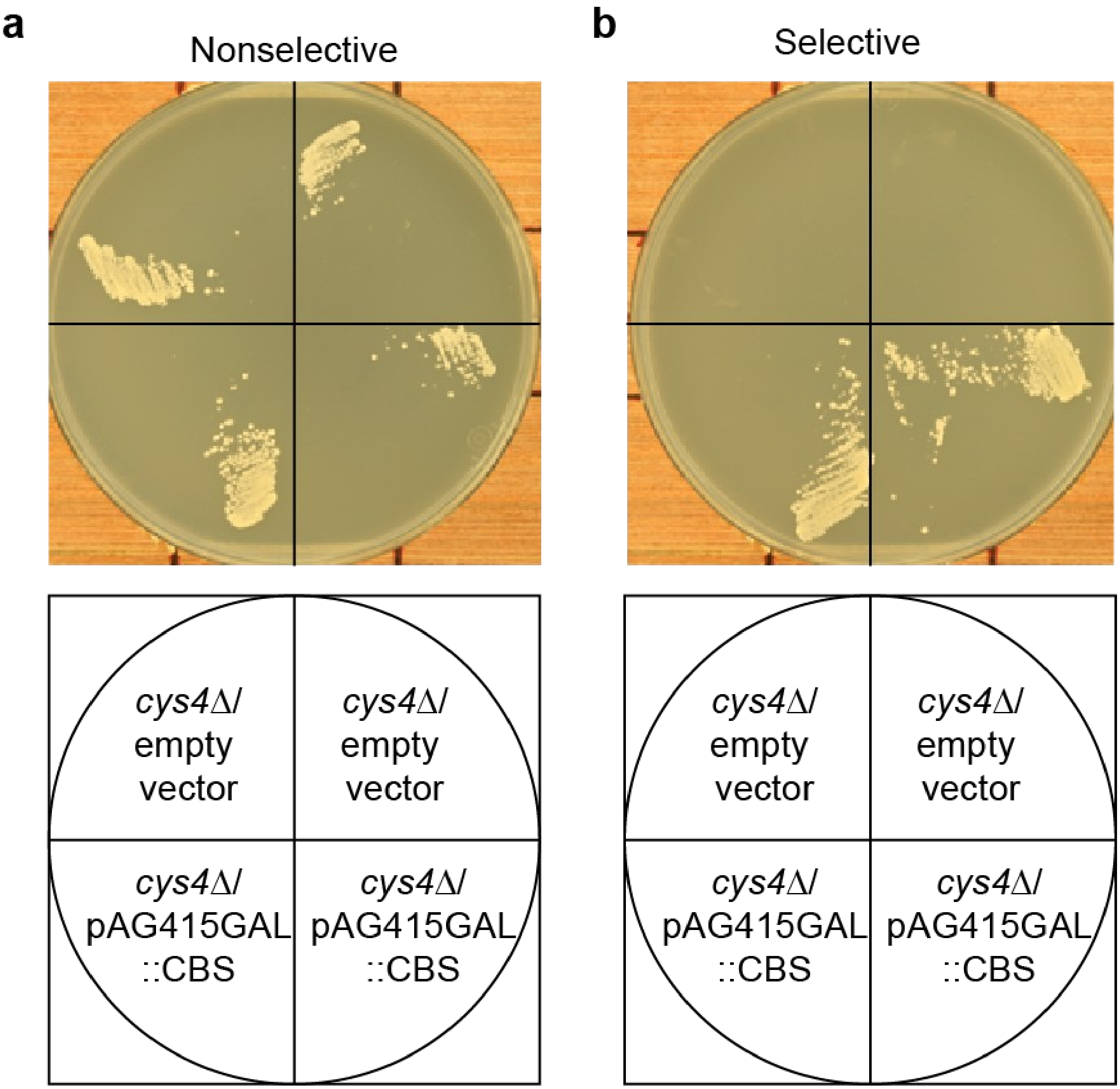

Figure S1. Expression of human CBS protein can rescue growth of yeast *cys4Δ* strain in the absence of exogenous cysteine. Growth of *cys4Δ* strain expressing *CBS* gene or carrying empty vector in non-selective (a) or selective (b) medium. The nonselective medium is synthetic complete medium lacking cysteine and supplemented with glutathione, a stable source of cysteine. The selective medium is synthetic complete medium lacking cysteine and glutathione. All medium was supplemented with galactose as carbon source to induce the expression.

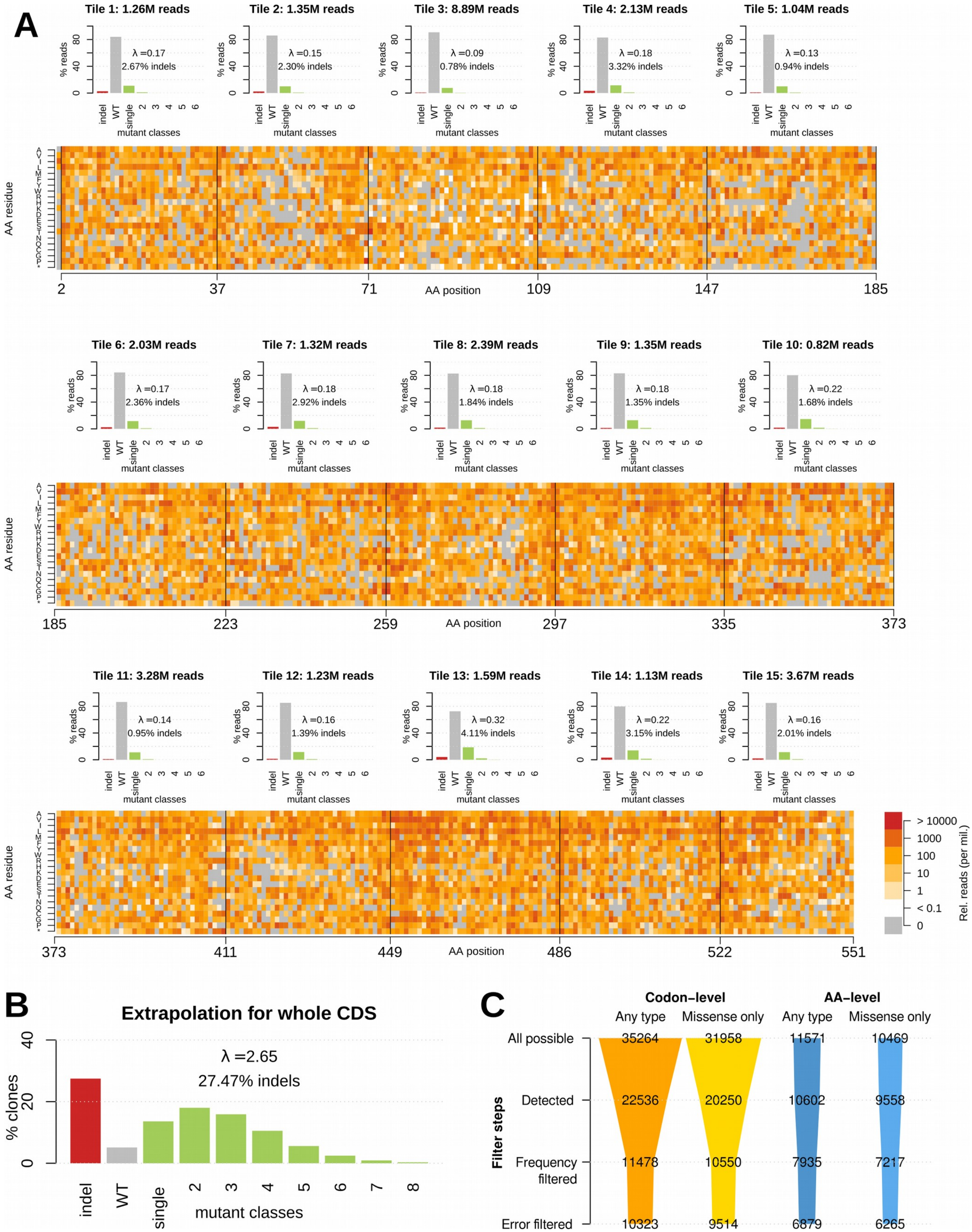

Figure S2: The CBS variant library underlying the VE maps is complex, but uniform. A: Each segment in the plot represents a sequencing tile along the CBS ORF. For each tile, a

histogram breakdown for the percentage of reads detecting indels, WT sequence, single, double or higher-level multi-mutants is shown. The lambda parameter corresponds to the best fit for a poisson distribution, indicating the estimated average number of mutations in the tile. The heatmap underneath shows relative frequency of reads detecting a particular codon change. Gray squares represent missing variants. The color scale stretches from white (< one in ten million reads) to dark orange (> 1 percent of reads).

B: An extrapolation of the number of mutations across the entire ORF based on the variants detected in each tile. The histogram shows the estimated percent of clones with insertions or deletions (indels), wildtype (WT) sequence, single-mutants, double-mutants and higher-level multi-mutants. The best fit is a Poisson distribution with an average number of 2.65 mutations per clone.

C: The effects of quality filtering on the number of variants in the low-vitamin B_6 dataset. The four columns show the number of total variants (i.e. synonymous, nonsense, and missense) at the codon level; missense-only variants at the codon-level; total variants at the amino acid level; and missense-only variants at the amino acid level, respectively. The four rows correspond to the different stages of processing: All theoretically possible variants; All variants that were detected in the non-selective condition with at least one sequencing read pair; All variants that passed the read frequency filtering step; and all variants that passed the regularized standard-error cutoff.

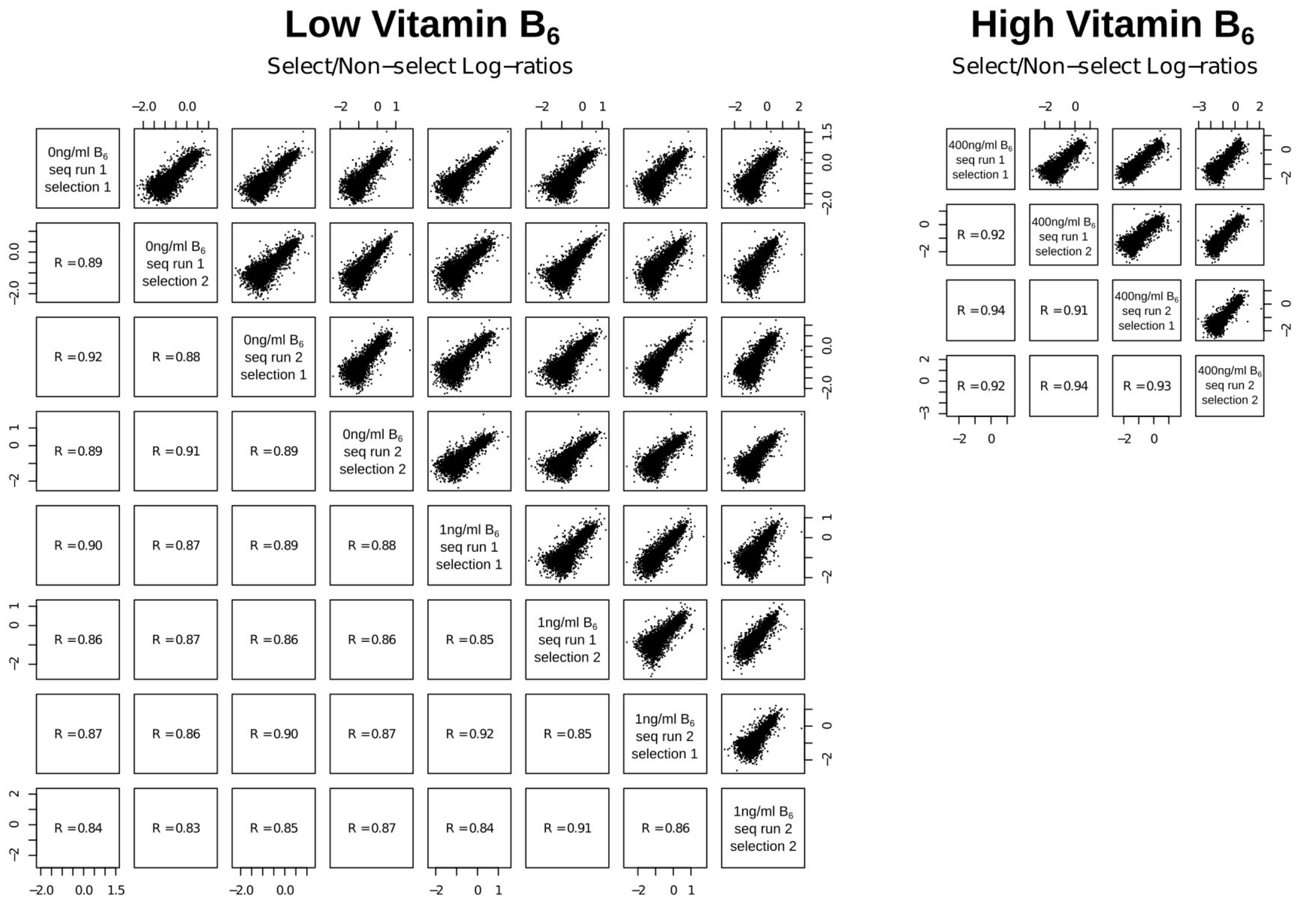

Figure S3: Correlations of technical replicates for the low vitamin B6 and high vitamin B6 VE map at the level of enrichment scores (i.e. the log ratio of relative allele frequencies in the post-selection vs pre-selection conditions). Diagonal fields indicate the respective replicate number. Tracing the x and y positions of each scatterplot or Pearson Correlation coefficient to the diagonal indicates which two replicates are being compared. The low vitamin B6 map encompasses measurements at 0ng/ml and 1ng/ml vitamin B6, (which upon discovering their virtual indistinguishability were treated as replicates of each other) thus featuring 8 instead of 4 replicates.

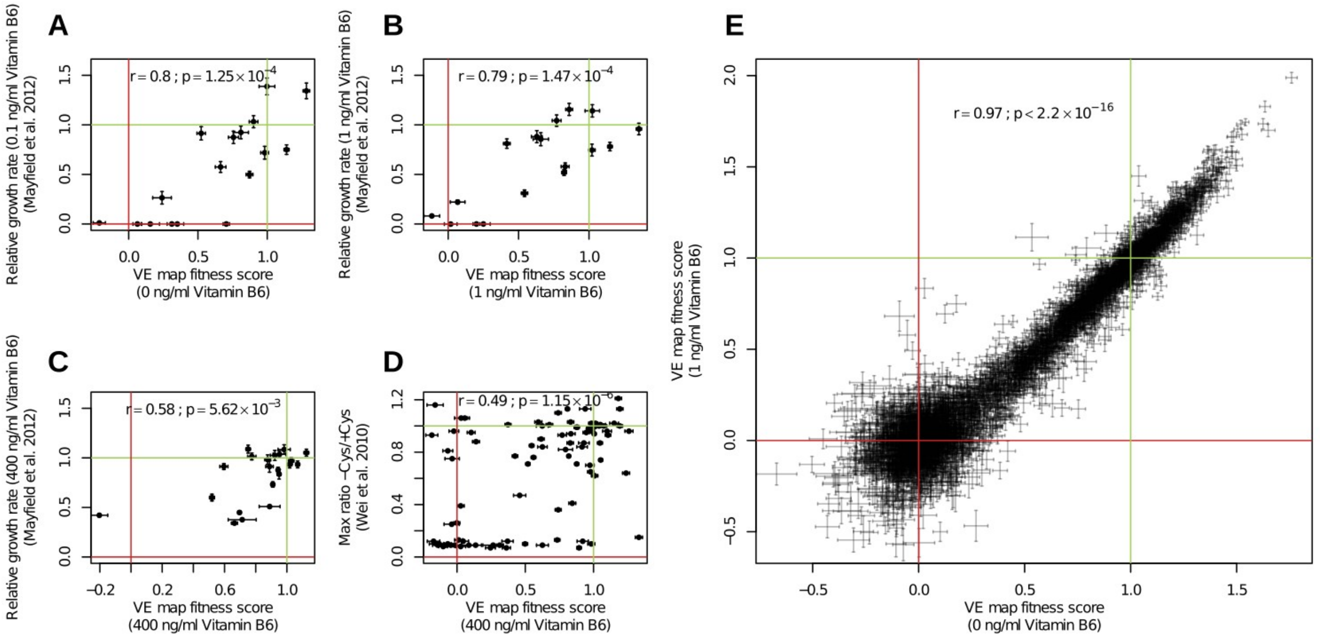

Figure S4. VE map fitness scores compared to relative growth rates determined in single-variant assays and strong correlation between VE map fitness scores with low concentrations of vitamin B6. (A-C) Correlation between (experimentally determined) fitness scores from VE maps and relative growth rates from single-variant assays at three different vitamin B6 concentrations. (D) Correlation between fitness scores from high vitamin B6 VE map and Cys-/Cys+ growth ratios from single-variant assays. (E) Correlation between VE map fitness scores with 0 and 1 ng/ml vitamin B6. The correlation test is Pearson correlation. Error bars represent standard error of the mean.

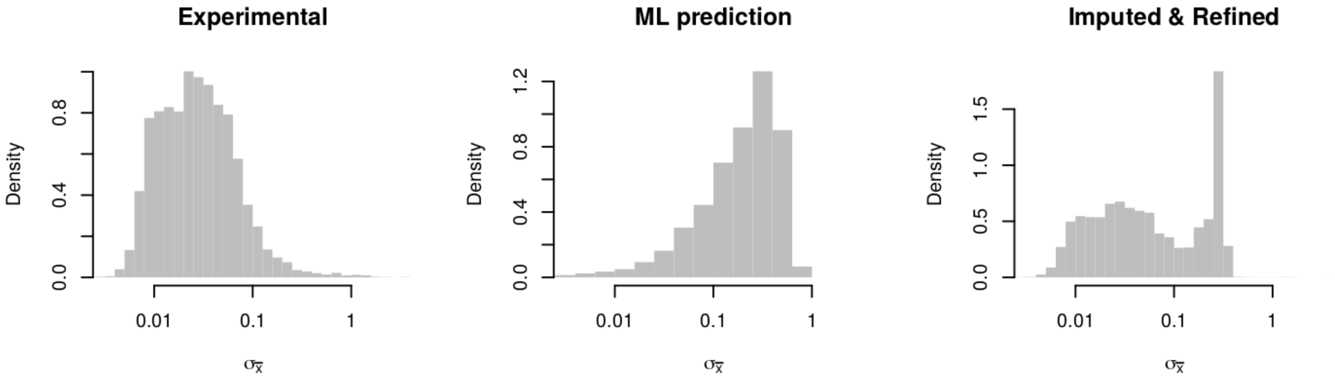

Figure S5: Distribution of standard error across dataset. Left: Distribution of Bayesian regularized standard error (see Methods) in the experimental data. Center: Distribution of predicted standard error in the machine learning predictions that form the basis of imputation and refinement. Right: Distribution of joint estimate of standard error in the refined and imputed dataset, i.e. confidence-weighted averages between experimental measurements and machine learning predictions.

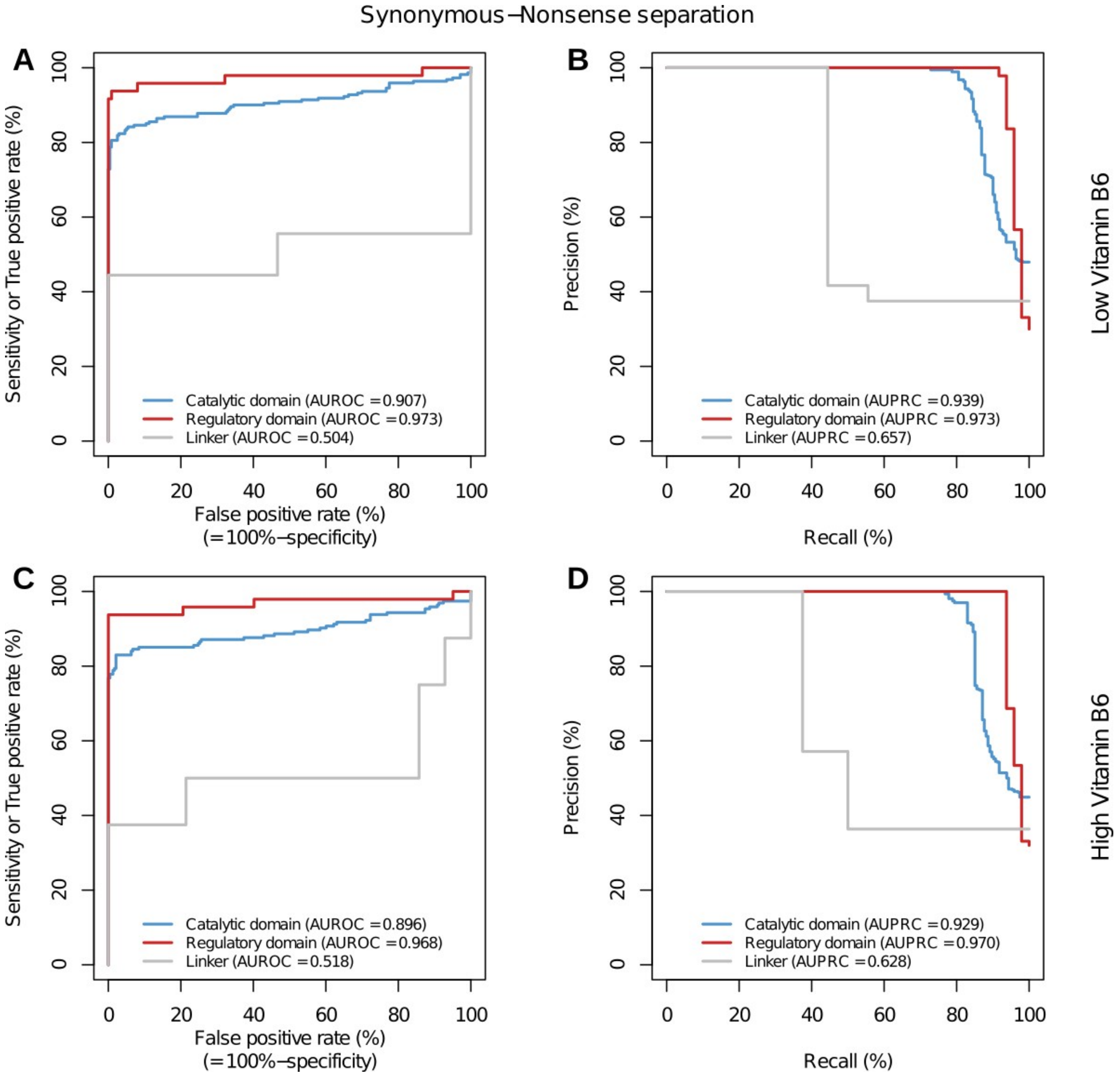

Figure S6: Receiver-Operator Characteristics (ROC) and Precision-Recall Curves (PRC) with respect to the maps ability to distinguish nonsense from synonymous variants in the catalytic domain, linker region and regulatory domain. (A) ROC curve for the low vitamin B6 VE map; (B) PRC curve for the low vitamin B6 map; (C) ROC curve for the high vitamin B6 VE map; and (D) PRC curve for the high vitamin B6 map.

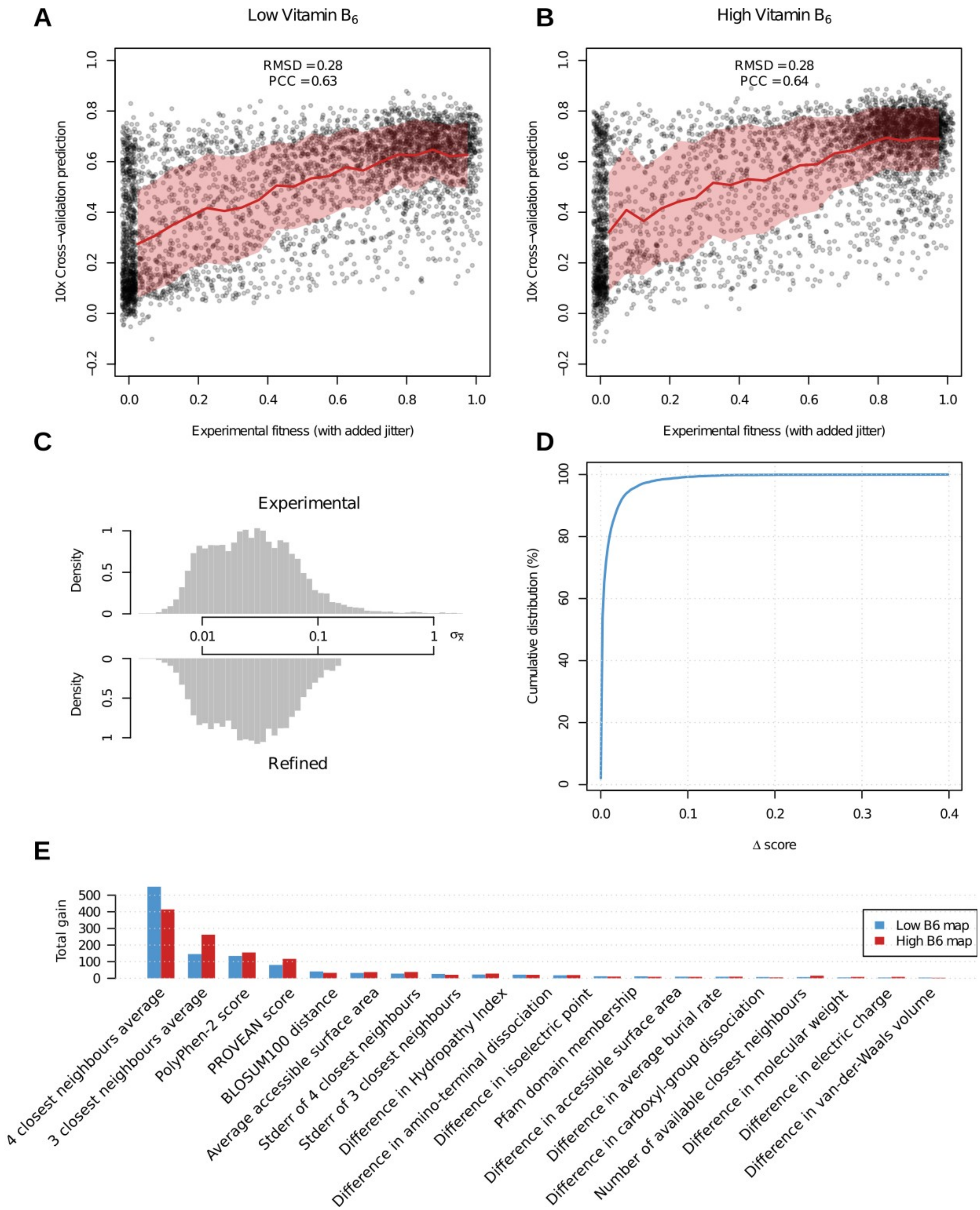

Figure S7: Evaluation of the imputation and refinement procedure. (A-B) 10x cross-validation of the machine learning predictions for the low- and high-vitamin B6 maps, respectively. The red line and shaded area indicate a moving-window average and standard deviation around the average). Uniformly distributed jitter [-0.01;0.01] was added to the x-axis. RMSD = Root-

mean-squared deviation. PCC = Pearson's Correlation Coefficient (C) Comparison of the distribution of standard error values for all measurements in the map before and after refinement (i.e. confidence-weighted averaging of measured and predicted values). (D) Cumulative distribution of changes to fitness scores as the result of refinement. (E) The twenty most important features used by the machine learning method.

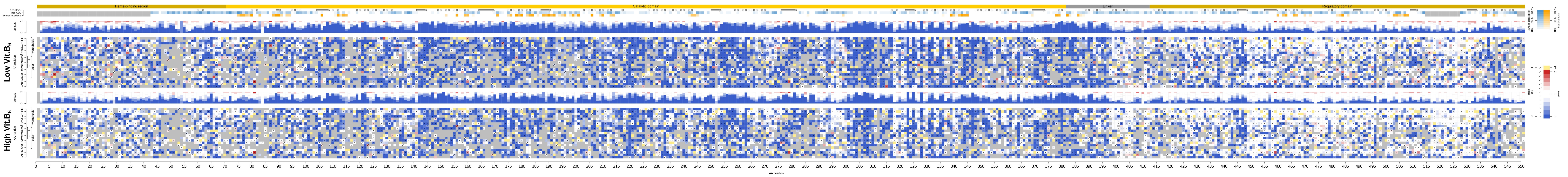

Figure S8: VE maps for CBS before computational imputation and refinement: fitness landscape with low level of vitamin B6 (top),fitness landscape with high level of vitamin B6 (middle) . For the two fitness landscapes (high or low vitamin B6), a functional score of 0 (blue) corresponds to a fitness equivalent to the median fitness of stop codon variants. A score of 1 (white) corresponds to a fitness equivalent to the median fitness of synonymous variants. A score greater than 1 (red) corresponds to fitness above the median fitness of synonymous variants.

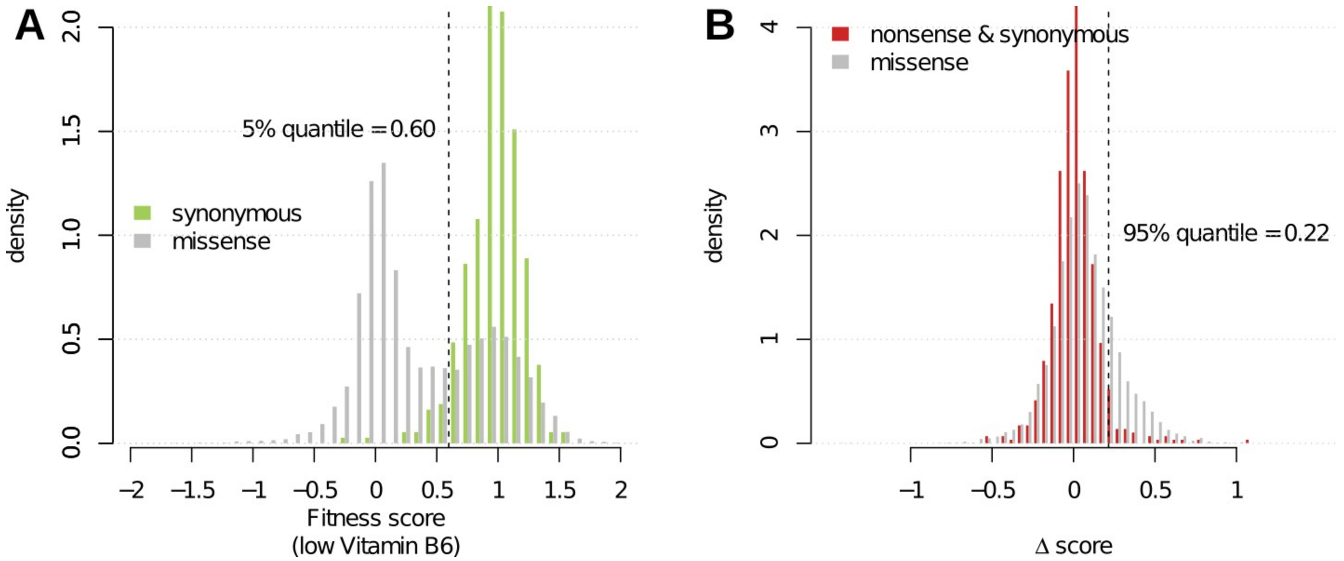

Figure S9. Determination of fitness score and delta fitness score cutoff values for significant fitness defect (0.60) and vitamin B6 remediability (0.22). (A) Classification of deleterious CBS variants with significant fitness defect using (imputed and refined) fitness score (low vitamin B_6) distribution of synonymous variants as the null distribution. (B) Classification of vitamin B_6-remediable deleterious variants using delta fitness (high-low vitamin B_6) distribution of stop codon variants as the null distribution.

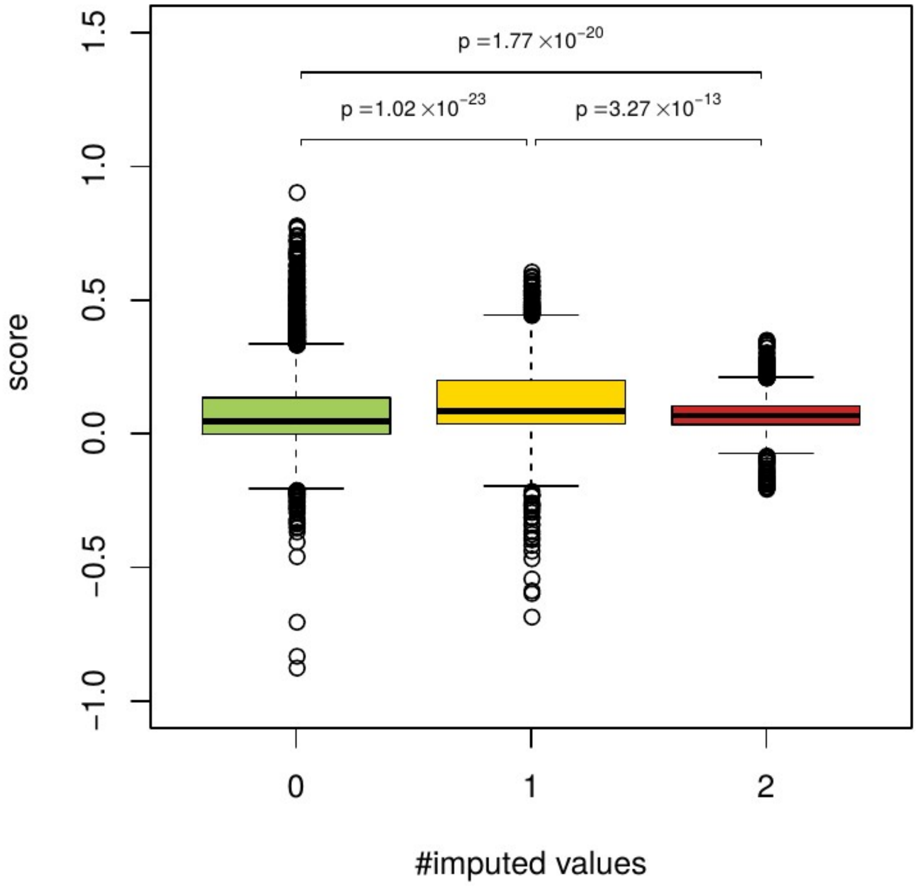

Figure S10: Distributions of delta scores (high vitamin B6 minus low vitamin B6) of variants for which none, one or both underlying scores are imputed. P-values correspond to Mann-Whitney U tests. Boxes indicate interquartile range, with bold horizontal lines indicating medians. Whiskers indicate maxima and minima inside 1.5x interquartile range.

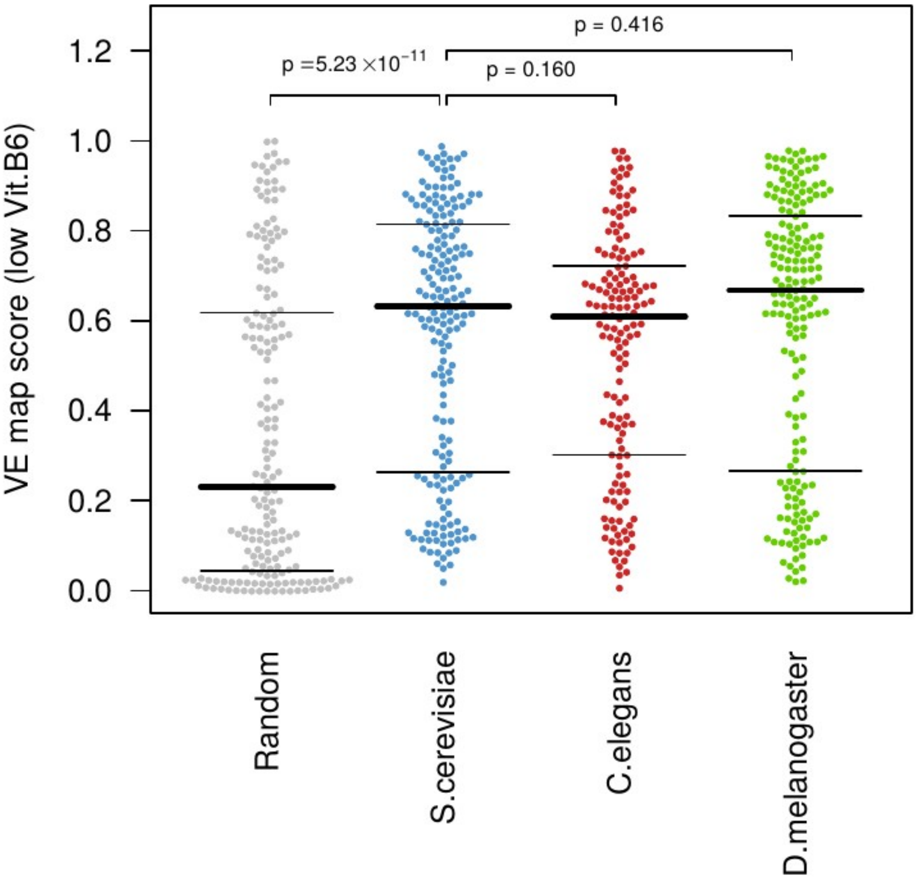

Figure S11: Distributions of VE map scores (in the low B6 condition) for variants corresponding to amino acids occurring in S.cerevisiae (yeast), C.elegans (worm) and D.melanogaster (fly), as well as randomly selected variants. P-values correspond to Mann-Whitney U tests. Bold horizontal bars correspond to medians, while thin horizontal bars indicate quartiles.

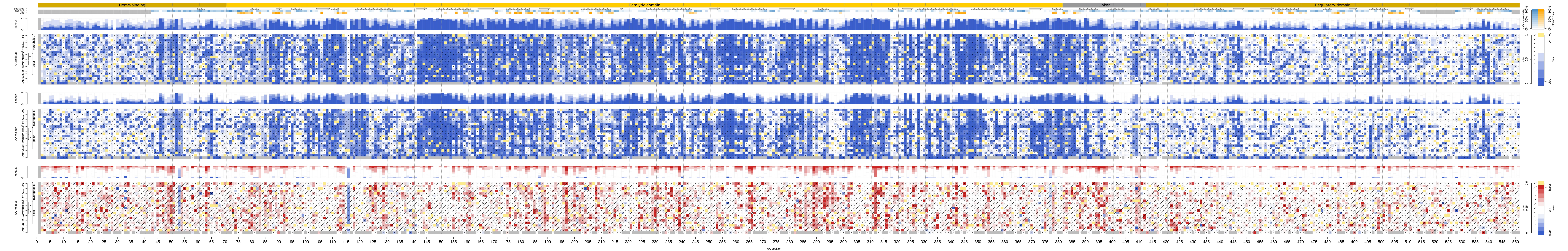

Figure S12: Full-length CBS variant effect (VE) maps. Fitness landscapes with low level (top) and high levels (center) of vitamin B6, and the delta fitness (high-low vitamin B6) landscape (bottom). The first four rows indicate domain annotations, secondary structure, relative solvent accessibility, and burial in the quaternary structure, respectively. The stacked histogram in each of the three plots summarizes the distribution of fitness scores at each amino acid position. Each main heatmap shows fitness scores for each possible amino acid substitution and nonsense mutation. For high and low vitamin B6 VE maps, a functional score of 0 (blue) corresponds to the median fitness of nonsense variants. A score of 1 (white) corresponds to the median fitness of synonymous variants. Yellow fields indicate the WT amino acid at each position. Gray fields indicate missing data. Diagonal lines indicate standard error, with crossed out fields marking variants for which standard error exceeded 1. For the delta fitness landscape (high-low vitamin B6), substitutions were colored red if delta fitness score is positive and blue if negative.

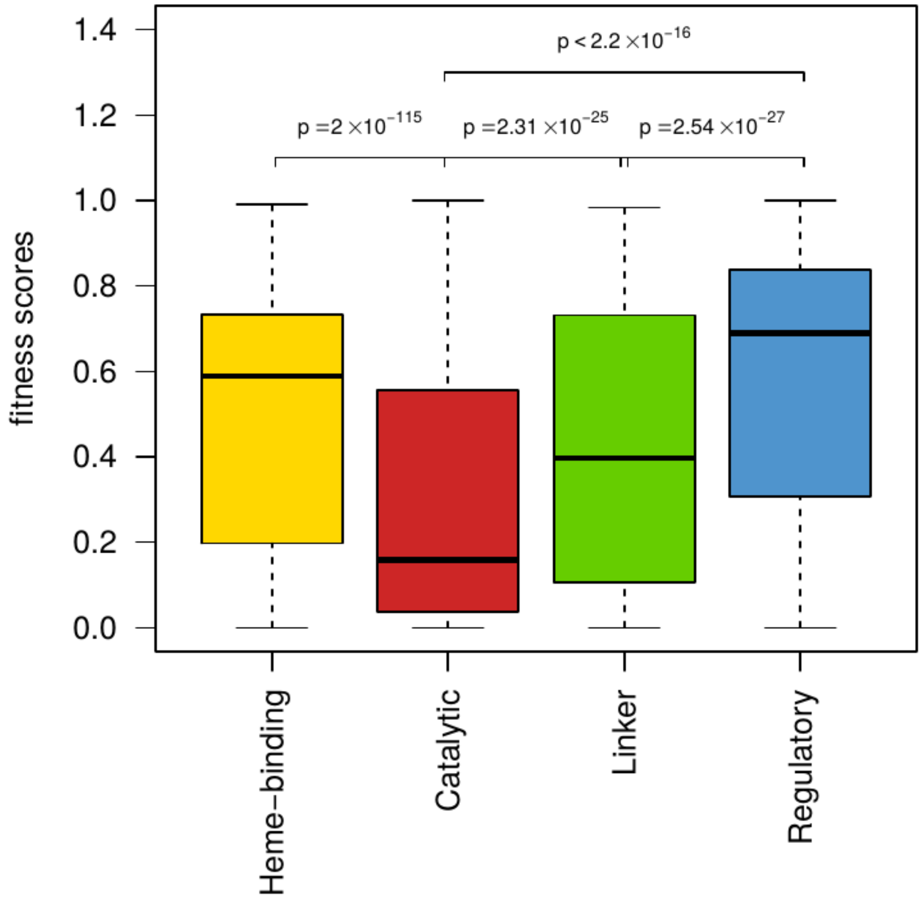

Figure S13: Distributions of (imputed and refined) VE map scores in the heme-binding domain, catalytic domain, linker region, and regulatory domain. Boxes correspond to interquartile range, with bold bars indicating medians. Whiskers correspond to minima and maxima. P-values correspond to Mann-Whitney U tests.

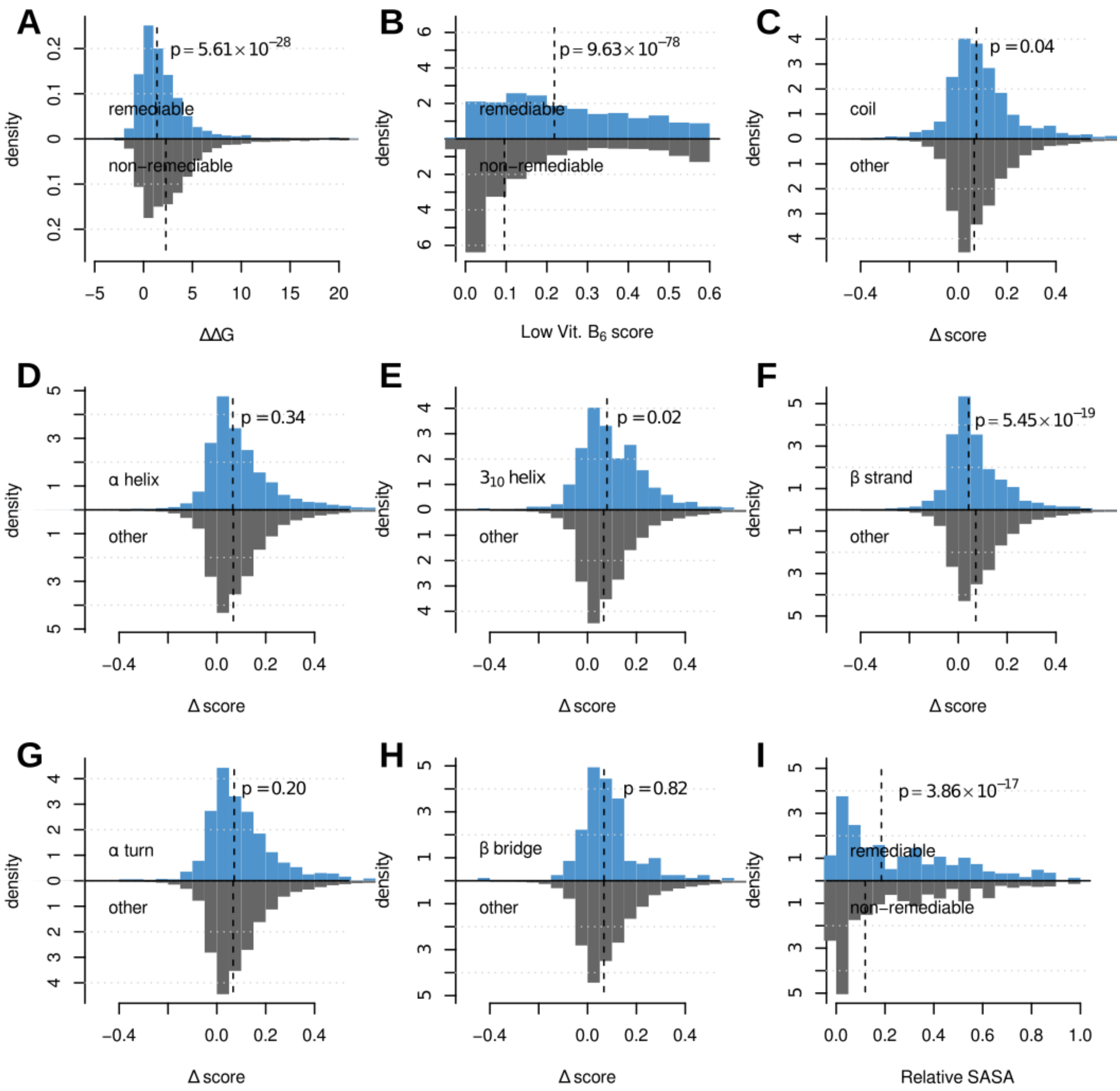

Figure S14. Vitamin B_6-remediable variants tend to have non-beta-strand secondary structures, higher solvent accessibility, smaller change in folding energy and higher fitness score in low vitamin B_6 map. Feature analysis for vitamin B_6 remediability of CBS variants. A) Comparison of change in folding free energy change between remediable and non-remediable variants. B) Comparison of fitness scores in the low B6 condition between remediable and non-remediable variants. C-H) Comparison of delta fitness score distributions between residues in different secondary structures: C) coil/non-coil; D) α-helix/non-α-helix; E) 3_{10}-helix/non-3_{10}-helix; F) β-strand/non-β-strand; G) α-turn/non-α-turn; H) β-bridge/non-β-bridge. I) Comparison of relative solvent-accessible surface area (SASA) between remediable and non-remediable variants.

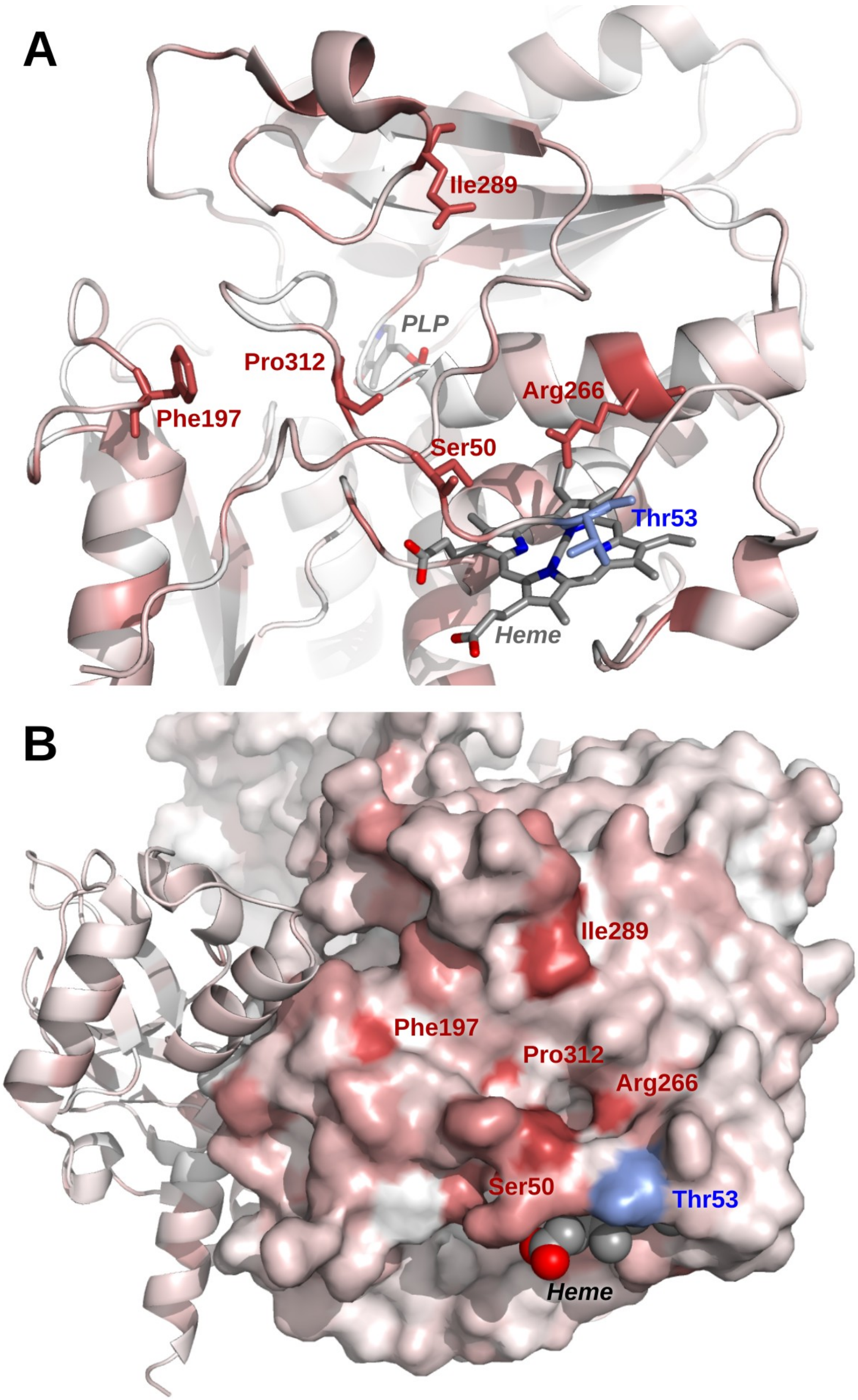

Figure S15: Structural context of the top 5 remediable residues. A) Cartoon view of CBS crystal structure in basal (inhibited) conformation (PDB entry 4L3V [17]) showing the top 5 residues with the highest median delta scores (labeled in red). Thr53 is labeled in blue,

featuring the second-lowest median delta score and being in physical proximity. Residues are colored according to median delta score, with red corresponding to positive, white to zero and blue to negative delta scores. B) Surface view of the same residues with colors as in A. The regulatory domain of a dimer binding partner is displayed in cartoon mode.

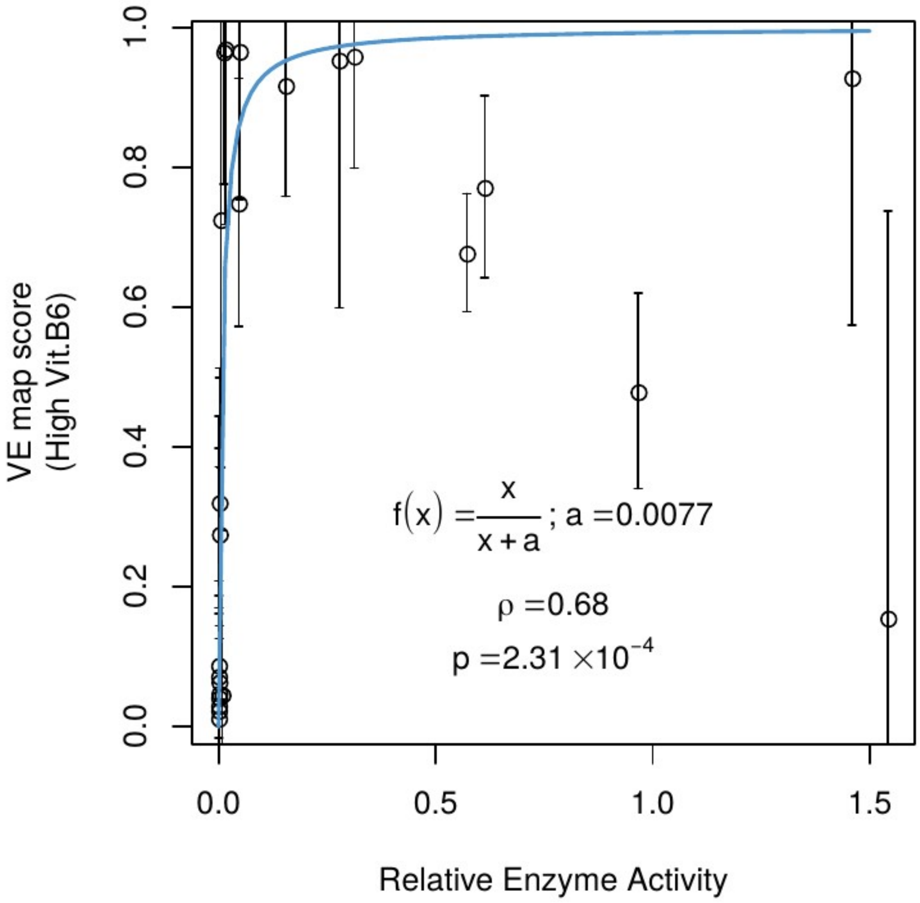

Figure S16: CBS relative enzyme activity (variant activity divided by wild type activity) correlated with VE map scores. The blue line shows the best maximum likelihood model for the non-linear relationship expected for recessive genes, i.e. y = x/(x+a), where y is fitness score, x is the enzyme activity. The maximum likelihood was achieved for a = 0.0077. The rho and p-value indicated correspond to Spearman’s rank correlation. Whiskers indicate regularized standard error.

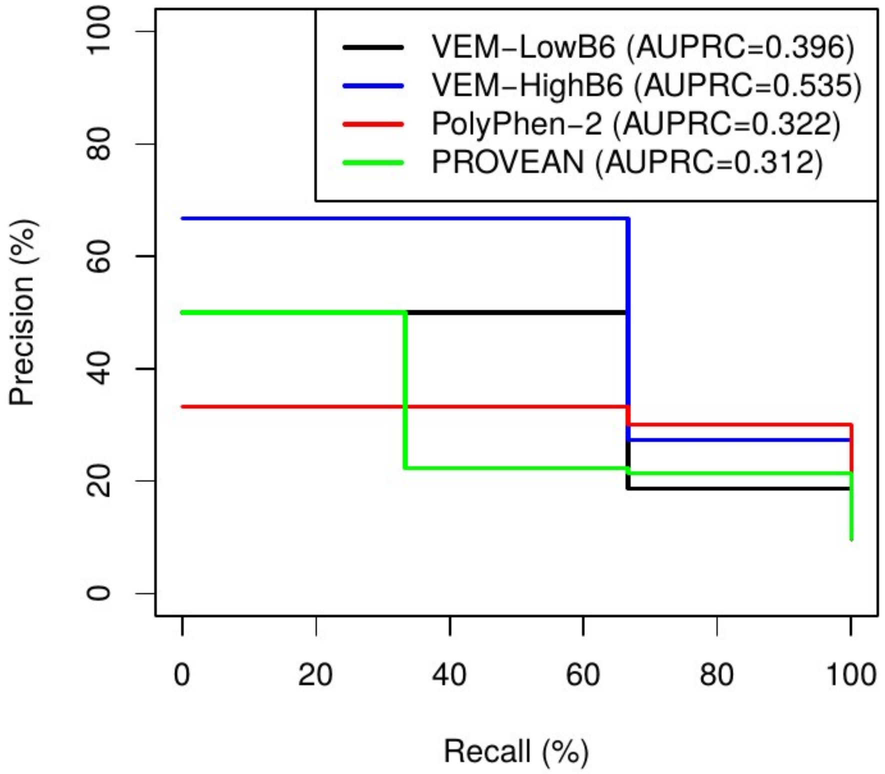

Figure S17: Precision-recall curve (PRC) for variants in the regulatory domain with respect to distinguishing three known positive reference disease variants against a gnomAD-based negative reference set. Black: Low Vitamin B6 VE map (imputed and refined); blue: High Vitamin B6 VE map (imputed and refined); Red: PolyPhen-2; Green: PROVEAN. AUPRC indicates area under the precision-recall curve.

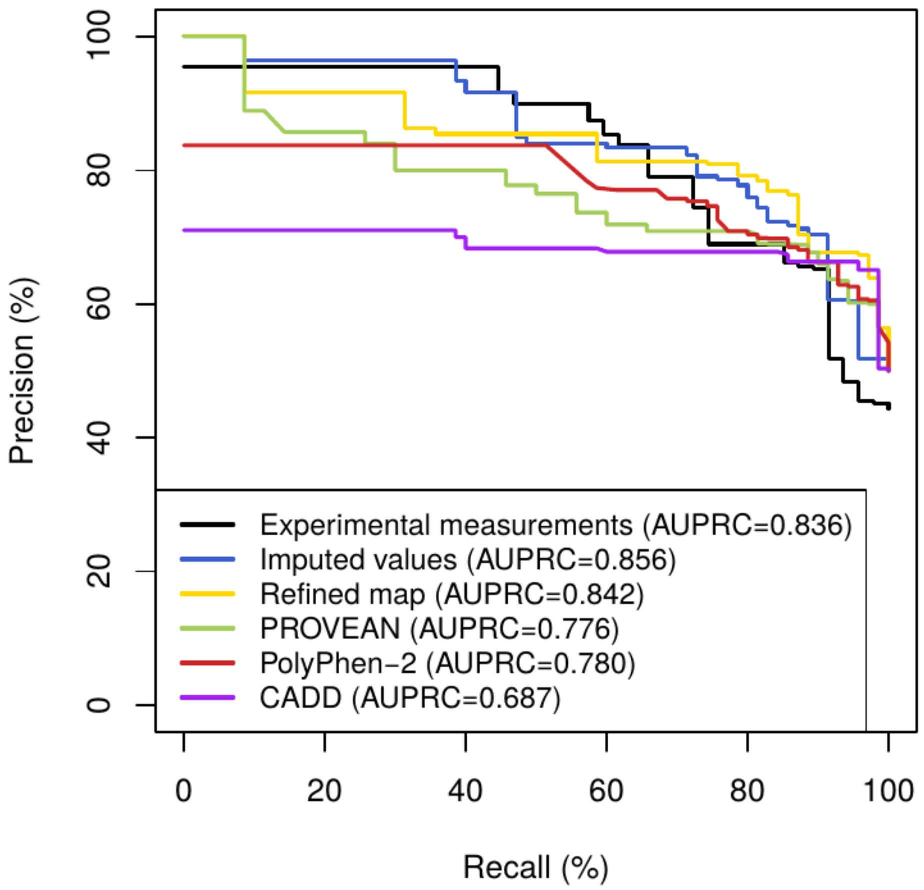

Figure S18: Precision-Recall curve (PRC) with respect to distinguishing known positive reference disease variants against the gnomAD-based negative reference set for raw experimental variant effect scores (black), raw machine learning predictions (blue), and final refined values (i.e. confidence-weighted averages between experimental scores and machine-learning predictions, yellow), as well as PROVEAN (green), PolyPhen-2 (red) and CADD (purple) predictions. AUPRC indicates area under the precision-recall curve.

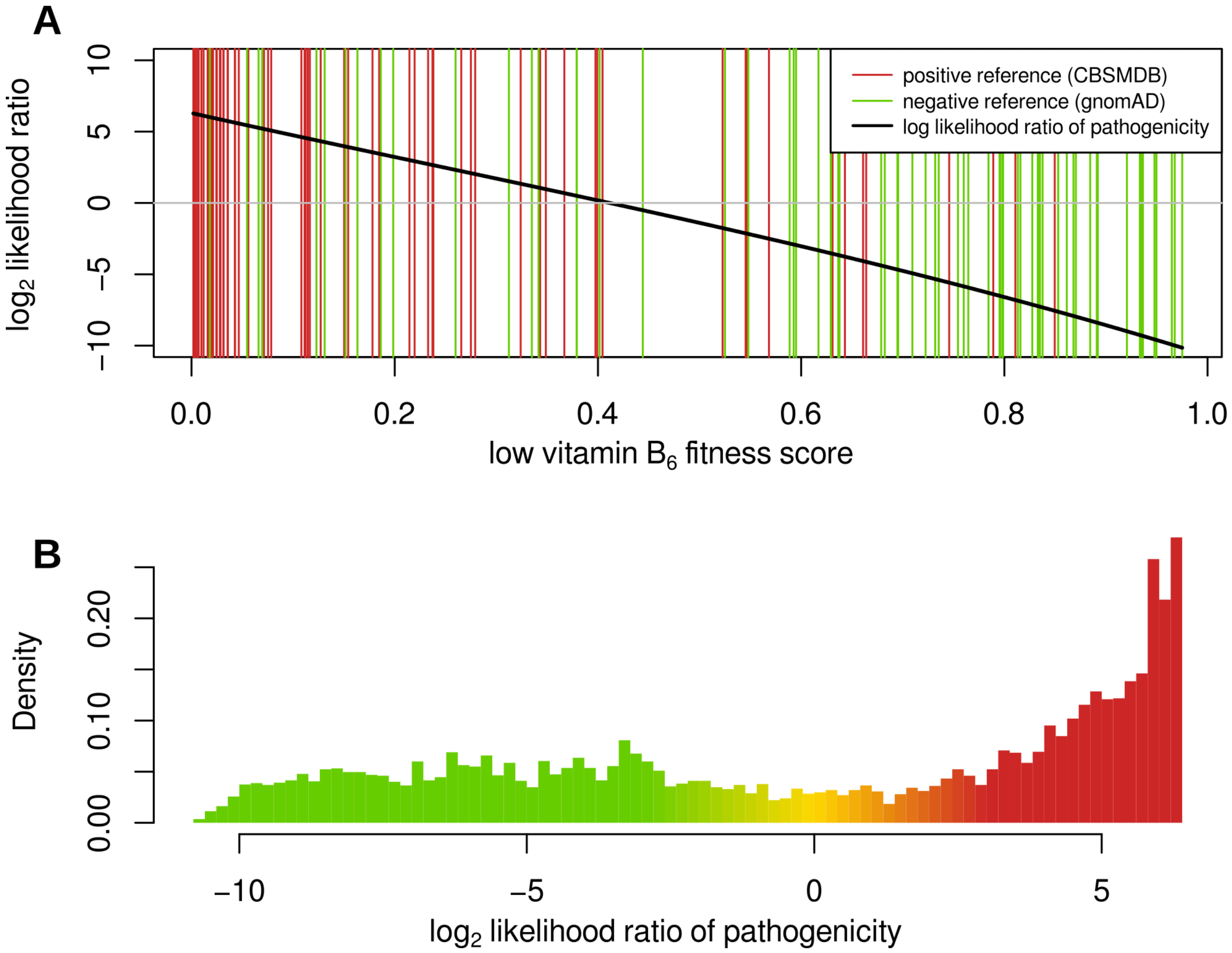

Figure S19: Calculation and distribution of log likelihood ratios (LLRs) of deleteriousness. Each score in a given map can be transformed into a log likelihood ratio of deleteriousness by calculating the log ratio between the probability of observing this score as part of the positive or negative reference variant sets. (A) Distribution of positive (red) and negative (green) reference variants in the low vitamin B6 map. The black line shows the corresponding LLR function. (B) The distribution of LLRs across all possible amino acid changes in CBS. Negative LLRs (green) indicate variants being more likely to be neutral, while positive LLRs (red) indicate variants more likely to be deleterious. LLRs near zero (yellow) indicate variants that are near parity.

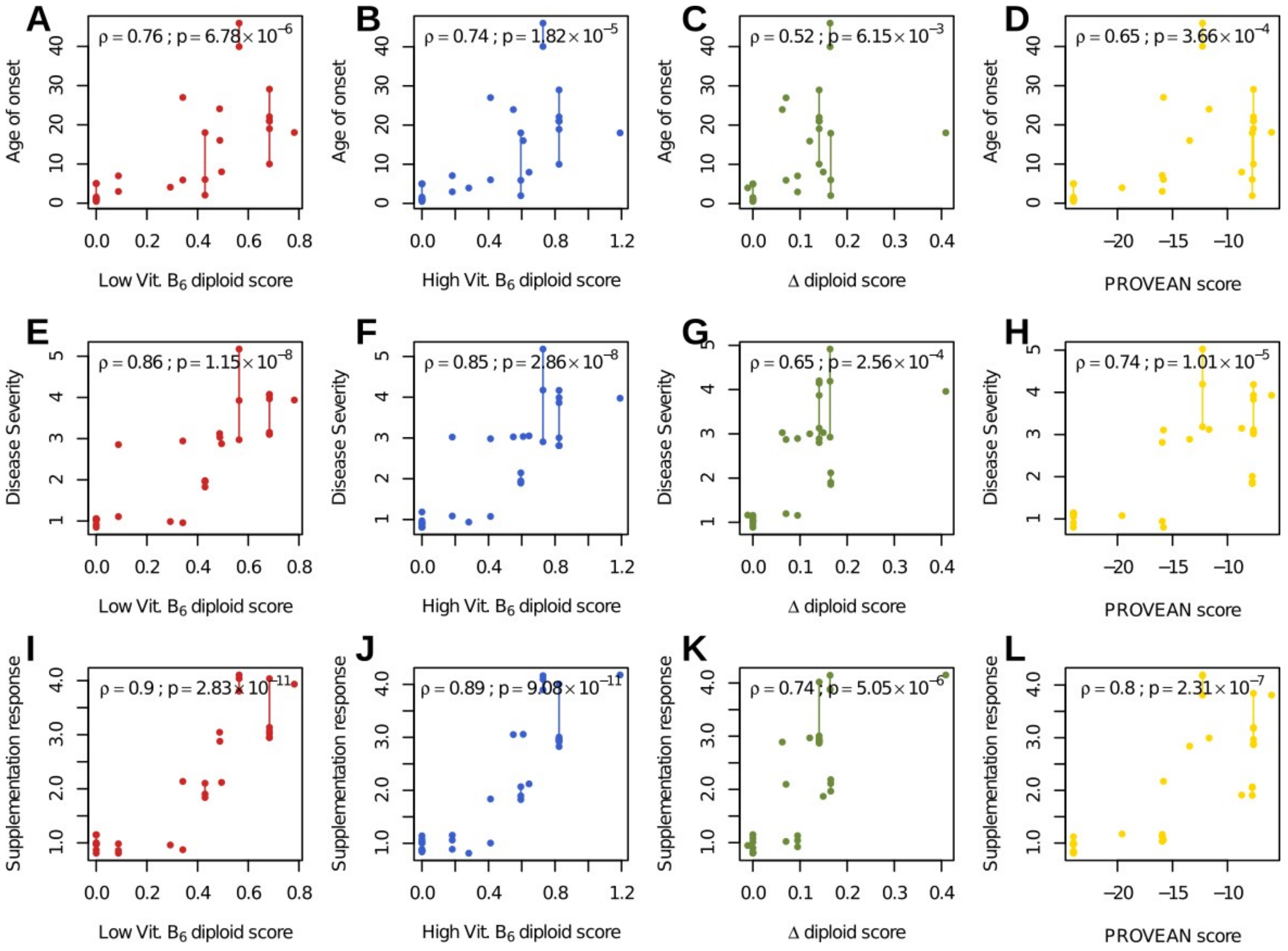

Figure S20. CBS diploid fitness scores based on the (imputed) VE maps successfully predict patient phenotype and response to vitamin B6 therapy, and outperform computational prediction. (A-D) Correlation between diploid VE map or PROVEAN scores and disease severity. (E-H) Correlation between diploid VE map or PROVEAN scores and age of onset. (I-L) Correlation between diploid VE map or PROVEAN scores and clinical B6 response. The correlation test is Spearman's Rank correlation. Degrees of disease severity: 5 = no symptoms at the time of diagnosis, 4 = mild disease, 3 = moderate disease, 2 = borderline severity, 1 = severe disease. Degrees of vitamin B6 responsiveness: 1 = nonresponsive, 2 = partial responsive, 3 = fully responsive, 4 = extremely pyridoxine responsive. A small amount of random noise ('jitter') was added to the categorical values of disease severity and vitamin B6 responsiveness to visually separate coincident data points. The amount of random noise is uniformly distributed in the interval [0;0.2]. Vertical lines connect cases with identical genotypes.

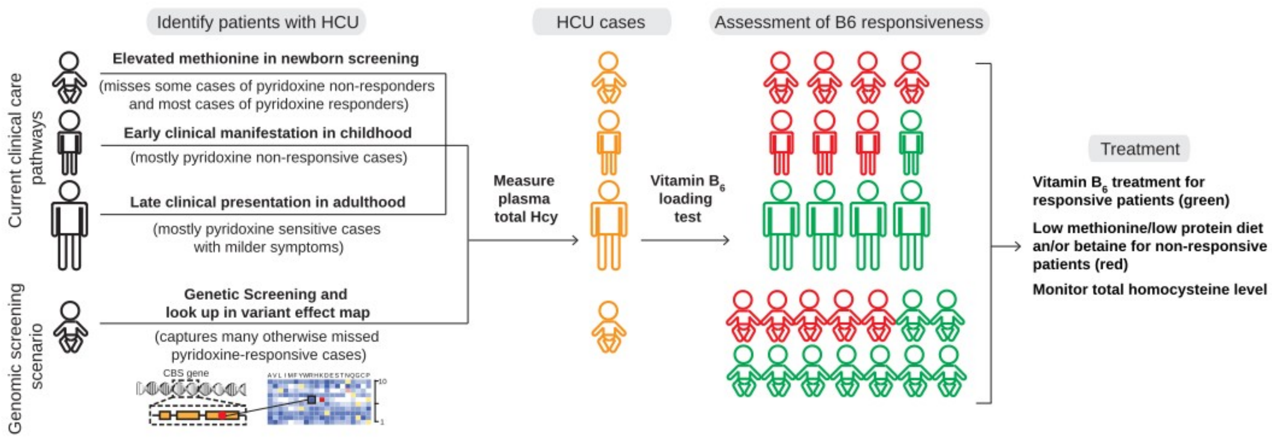

Figure S21: Diagnosis and treatment of homocystinuria (HCU) patients. Current clinical care pathways for identifying, confirming and treating homocystinuria patients (which probably miss many cases of CBS deficiency and especially those that are most responsive to vitamin B6 therapy) and a proposed genomic screening scenario.
